# Mathematical model analysis and numerical simulation for codynamics of meningitis and pneumonia infection with intervention

**DOI:** 10.1038/s41598-022-06253-0

**Published:** 2022-02-16

**Authors:** Belela Samuel kotola, Temesgen Tibebu Mekonnen

**Affiliations:** Department of Mathematics, Debre Berhan University, Debre Berhan, Ethiopia

**Keywords:** Computational biology and bioinformatics, Health care, Mathematics and computing

## Abstract

In this paper, we have considered a deterministic mathematical model to analyze effective interventions for meningitis and pneumonia coinfection as well as to make a rational recommendation to public healthy, policy or decision makers and programs implementers. We have introduced the epidemiology of infectious diseases, the epidemiology of meningitis, the epidemiology of pneumonia, and the epidemiology of infection of meningitis and pneumonia. The positivity and boundedness of the sated model was shown. Our model elucidate that, the disease free equilibrium points of each model are locally asymptotically stable if the corresponding reproduction numbers are less than one and globally asymptotically stable if the corresponding reproduction numbers are greater than one. Additionally, we have analyzed the existence and uniqueness of the endemic equilibrium point of each sub models, local stability and global stability of the endemic equilibrium points for each model. By using standard values of parameters we have obtained from different studies, we found that the effective reproduction numbers of meningitis ${\mathcal{R}}_{eff(m)}=9$ and effective reproduction numbers of pneumonia ${\mathcal{R}}_{eff(p)}=11$ that lead us to the effective reproduction number of the meningitis and pneumonia co-infected model is $max\left\{ {\mathcal{R}}_{eff\left(m\right)}, {\mathcal{R}}_{eff(p)} \right\}=9$. Applying sensitivity analysis, we identified the most influential parameters that can change the behavior of the solution of the meningitis pneumonia coinfection dynamical system are ${\alpha }_{1} , {\alpha }_{2}$ and $\pi$. Biologically, decrease in ${\alpha }_{1}$ and increasing in $\pi$ is a possible intervention strategy to reduce the infectious from communities. Finally, our numerical simulation has shown that vaccination against those diseases, reducing contact with infectious persons and treatment have the great effect on reduction of these silent killer diseases from the communities.

## Introduction

An infectious disease is illness which is clinically evident resulting from the presence of a pathogenic microbial agent, such as virus, bacterium, protozoa, or toxin, that can be passed from one host to another through modes of transmission such as direct physical contact, airborne droplets, water or food, disease vectors, or mother to newborn^1–4^. One of most common infectious microbial agent is *Streptococcus pneumoniae* bacterium, which is responsible for pneumococcal disease such as pneumonia, meningitis, and sepsis^5^.

Specifically, Cerebrospinal meningitis (CSM) is a dangerous disease caused by Neisseria meningitis (meningococcal), which colonizes the nasopharynx (the area of the upper throat that lies behind the nose) and spreads when an individual encounters infected respiratory secretions^6^. From our body part, the lung is exposed to approximately 10,000 L of air per day, which may contain infectious or toxic agents’ leads to pneumonia disease^3^.

Pneumonia is one of the forgotten killer but, treatable respiratory lung infectious diseases caused by bacteria, fungi, virus or parasites^7–9^. The most common cause of bacterial pneumonia and remains a substantial source of morbidity and mortality in both developing and developed countries is *Streptococcus pneumonia*^10,11^. *Streptococcus pneumonia* also known as pneumococcus pneumonia, which is characterized primarily by inflammation in the air sacs (alveoli) in the lungs that are filled with fluid or pus, making it difficult to breathe and is a form of acute respiratory tract infection (ARTI) that affects the lungs^10–14^.

Co-infected is the process of infection of a single host with two or more pathogen variants (strains) or with two or more distinct pathogen species. Coinfection with multiple pathogen strains is common in pneumonia; nevertheless, it occurs in many other diseases. Super infection is defined as infection with a second strain after the initial infection, and the immune response to it has been established^15,16^.

Mathematical models for transmission dynamics of any diseases are mandatory in providing better insights into the behavior of the disease, allowing us to optimize use of limited resources, and recommending the control measures on the infectious disease^17^. The decision made regarding to intervention strategies for preventing and controlling the insurgence of pneumonia and meningitis are successful where, it can be influenced by developed model on these diseases^18^. The emerging and reemerging of the diseases makes mathematical modeling very useful and attractive area for making estimation on the impact of a control measure and of underlying parameters of a real-world phenomenon, which are difficult or expensive to obtain through experiment^18^.

We had reviewed some selected literatures of mathematical models done on either Meningitis infection, Pneumonia infection, as well as Meningitis and Pneumonia Co-infection. Moreover, we had used them as basis for our developed model on Meningitis and Pneumonia Co-infection.

Different authors have studied the coinfection of various diseases, such as^19^, who stated the coinfection of HIV and pneumonia and^20^ provided the coinfection of pneumonia and malaria. There is also the research on HIV/AIDS-pneumonia coinfection model with treatment at each infection stage with corresponding numerical simulations using mat lab software studded by^9^. Moreover^21^, discussed the mathematical modelling of influenza-meningitis under the quarantine and^22^, invested their effort on a mathematical model for co-dynamics of listeriosis and bacterial meningitis diseases. However, very few researchers have investigated coinfection of pneumonia and meningitis. Moreover, no one consider the mathematical model for pneumonia and meningitis infection with a single vaccination class for both disease simultaneously. There are immunization called PCV13, that can protects against 13 types of pneumococcal bacteria, which cause the most common pneumococcal infections in kids and immunization called PPSV23, which can protects against 23 types^5^. One of the primary reasons of our work is to fill this gap and making sound recommendation to public healthy, policy decision makers or programs implementers.

## Mathematical model

### Basic assumptions and description of parameters

In this section, we presented the mathematical model of meningitis-pneumonia coinfection by considering a homogenous population (i.e. in which every person has the same chance of coming in contact with an infected person), and factors such as sex, social status, and race do not affect the probability of being infected. The model subdivides the human total population $N(t),$ into seven mutually exclusive compartments, namely, susceptible population $S(t),$ pneumonia only infectious ${I}_{p}(\mathrm{t}),$ meningitis only infectious ${I}_{m}(\mathrm{t})$, meningitis and pneumonia co infectious ${I}_{mp}(\mathrm{t}),$ meningitis, pneumonia co-infected treated class ${T}_{mp}(t$), vaccinated (PCV Pneumococcal conjugate vaccine) group ${V}_{mp}(t$) and recovered $(\mathrm{R})$. The vaccinated class (${V}_{mp}\left(t\right)$) is the group of people those who took the vaccination called pneumococcal conjugate vaccine (PCV13) against invasive pneumococcal disease such as pneumonia and meningitis.

In this study, recovery from natural immunity is significant for pneumonia only infected individuals and meningitis-only infected individuals and then joins the recovered compartment, we denote such a natural recovery rate as ${\tau }_{1}$ and ${\tau }_{2}$ for meningitis and pneumonia, respectively. The effects of vertical transmission to pneumonia and meningitis were assumed insignificant in this study. From an epidemiological perspective, individuals in the removed/recovered compartment $R(t),$ do not attained permanent immunity. The mass action incidence rate of new infections was used in this study and the modification parameters $\upomega$ and $\Theta$ are the factors by which the infectiousness of pneumonia increases the susceptibility of meningitis and vice versa, respectively. Pneumonia and meningitis are assumed to be transmitted after effective contact between susceptible and infectious classes with effective contact rates ${\alpha }_{1}$ and ${\alpha }_{2}$, respectively. Individuals can develop meningitis by contact rate of ${\alpha }_{2}$ from meningitis only infected or co-infected person with force of infection of meningitis ${f}_{1} ={ \alpha }_{2}\left({I}_{m}+{I}_{mp}\right)$ and join the ${I}_{m}$ compartment. An individual can develop pneumonia with a contact rate of ${\alpha }_{1}$ from pneumonia-only infected or co-infected person with a force of infection of pneumonia ${f}_{2} ={\alpha }_{1}({I}_{p}+{I}_{mp})$ and then join ${I}_{p}$ compartment. Pneumonia-only infected individuals can also develop an additional meningitis infection with force of infection and modification parameters ${\upomega f}_{1}$ and join the co-infected compartment ${I}_{mp}$. The co-infected compartment increases because of individuals who come from meningitis-only infected compartments when they are infected by pneumonia with force of infection and modification parameters ${\Theta f}_{2}$.

The parameters used in the model are described in the table below.

Using the above basic model assumption and parameters described in Table 1, we have the following flow-chart.

**Table 1:** Descriptions of parameters.

Number	Parameter	Description
1	${\tau }_{1}$	The rate at which meningitis infected individual are recovered naturally
2	${\tau }_{2}$	The rate at which meningitis and pneumonia co infected individual treated and inter to treated class
3	$\beta$	The rate at which meningitis and pneumonia co infected individual are recovered from both diseases
4	$\mu$	Natural death rate
5	${\delta }_{1}$, ${\delta }_{2}$	Meningitis only caused death rate and Pneumonia only caused death rate, respectively
6	${\delta }_{3}$	Meningitis and pneumonia co-infected caused death rate
7	$\omega ,\Theta$	Modification parameter, where $\upomega \ge 1 \; and \; \Theta\ge 1$
8	$\rho$	Rate of loss of immunity
9	$\pi$	The portion of vaccinated new born
10	$\lambda$	Recruitment rate
11	${\alpha }_{2}$, ${\alpha }_{1}$	Meningitis and Pneumonia contract rate, respectively
12	$\phi$	vaccine wanes rate
13	${ \tau }_{3}$	The rate at which pneumonia infected individual are recovered naturally

**Figure 1:**
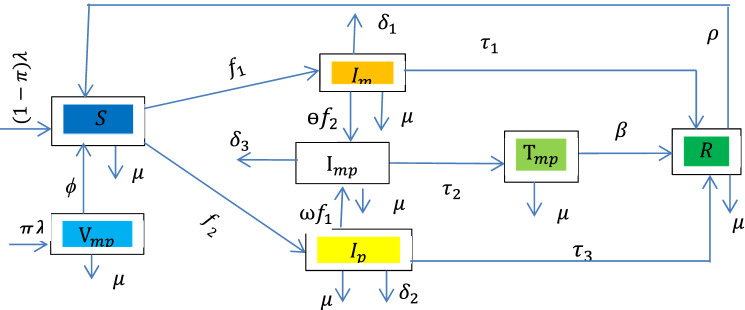
Flow chart of meningitis and pneumonia coinfection.

Using the parameters in Table 1 and the flow chart in Fig. 1, we have the following dynamical system

1 $$\left.\begin{array}{l}\frac{dS}{dt} =\left(1-\pi \right)\lambda +\rho R+\phi {V}_{mp}-\left({f}_{1}+{f}_{2} +\mu \right)S\\ \frac{d{I}_{P}}{dt}={f}_{2}S-\left(\upomega{f}_{1} +{\tau }_{3} +{\delta }_{2} +\mu \right){I}_{p} \\ \frac{d{I}_{m}}{dt}={f}_{1}S-\left({\Theta f}_{2}+ {\tau }_{1}+{\delta }_{1}+\mu \right){I}_{m} \\ \frac{d{I}_{mp}}{dt}={\upomega f}_{1}{I}_{p}+{\Theta f}_{2}{I}_{m}-\left({\tau }_{2}+\mu +{\delta }_{3}\right){I}_{mp} \\ \frac{d{\mathrm{T}}_{mp}}{dt}={\tau }_{2}{I}_{mp}-\left(\beta +\mu \right){\mathrm{T}}_{mp} \\ \frac{d{V}_{mp}}{dt}=\pi \lambda -\left(\upmu +\phi \right){V}_{mp} \\ \frac{d\mathrm{R}}{dt}=\beta {T}_{mp}+{\tau }_{1}{I}_{m}+{\tau }_{3}{I}_{P}-\left(\rho +\mu \right)R \end{array}\right\}$$

## Qualitative analysis of the model

In this section, we have presented the qualitative behavior of the model. For simplification of mathematical manipulations, we split the full meningitis-pneumonia confection model into sub-models as meningitis only model and pneumonia only model. The qualitative behavior of the sub-models is studied first, and the qualitative behavior of the full model then follows.

### Meningitis only model

To gate meningitis only model from the full model (1), we set, ${I}_{p}={I}_{mp}={T}_{mp}=0$ and we do have the following dynamical system,

2 $$\left.\begin{array}{c}\frac{dS}{dt}=\left(1-\pi \right)\lambda +\rho R+\phi {V}_{m}-\left({f}_{1}+\mu \right)S \\ \frac{d{I}_{m}}{dt}={f}_{1}S-\left( {\tau }_{1}+{\delta }_{1}+\mu \right){I}_{m} \\ \frac{d{V}_{m}}{dt}=\pi \lambda -\left(\upmu +\phi \right){V}_{m} \\ \frac{d\mathrm{R}}{dt}={\tau }_{1}{I}_{m}-\left(\rho +\mu \right)R \end{array} \right\}$$

#### Positivity of solutions of the meningitis only model

To be assure that the developed dynamical system (2) is epidemiologically meaningful and posed, we need to prove that all the state variables of dynamical systems are nonnegative.

##### **Theorem 1**

*All the populations of the system with positive initial conditions are nonnegative*.

##### *Proof*

Assume $S\left(0\right)>0 , {I}_{m}\left(0\right)>0, {V}_{m}\left(0\right)>0 \; and \; R\left(0\right)>0$ are positive for time $t>0$ and for all nonnegative parameters. Let $T=\mathrm{sup}\{t>0 \; such \; that \; S\left({t}^{{\prime}}\right)>0 , {I}_{m}\left({t}^{{\prime}}\right)>0, {V}_{m}\left({t}^{{\prime}}\right)>0 \; and \; R\left({t}^{{\prime}}\right)>0 , {t}^{{\prime}}\in [0, t] \}$

From the initial condition, all the state variables are nonnegative at the initial time; then, $T>0$

From fist equation of system (2) $\frac{dS}{dt}=\left(1-\pi \right)\lambda +\rho R+\phi {V}_{m}-\left({f}_{1}+\mu \right)S$

$$\frac{dS}{dt}+\left({f}_{1}+\mu \right)S=\left(1-\pi \right)\lambda +\rho R+\phi {V}_{m}$$

$$\frac{d}{dt}\left[{e}^{{\int }_{0}^{T}\left({f}_{1} +\mu \right)d{t}^{{\prime}}}S\right]={e}^{{\int }_{0}^{T}\left({f}_{1} +\mu \right)d{t}^{{\prime}}}[\left(1-\pi \right)\lambda +\rho R+\phi {V}_{m}]$$

$$\Rightarrow S\left(t\right)={k}_{1}S\left(0\right)+{k}_{1}\left[{\int }_{0}^{T}{e}^{{\int }_{0}^{T}\left({f}_{1} +\mu \right)d{t}^{{\prime}}}[\left(1-\pi \right)\lambda +\rho R+\phi {V}_{m}]dt\right]>0$$

where ${k}_{1}={e}^{-{\int }_{0}^{T}\left({f}_{1} +\mu \right)d{t}^{{\prime}}}$, which is non-negative because it is an exponential function.

$$\Rightarrow S\left(t\right)>0$$

Following the same procedure, we have ${I}_{m}\left(t\right)>0$ , ${V}_{m}\left(t\right)>0$ and $R\left(t\right)>0.$

Therefore, from the above proof, we can conclude that whenever the initial values of the systems are all nonnegative, then all the solutions of our dynamical system are positive.

#### Invariant region of the meningitis only model

##### **Theorem 2**

*The dynamical system* (2) *is positively invariant in the closed invariant set*

${\Omega }_{1}=\{\left(S, {I}_{m, } {V}_{m, }R\right)\epsilon {\mathbb{R}}_{+}^{4}:{ N}_{1}\le \frac{\lambda }{\mu }\}$, *where* ${N}_{1}$ *is the total human population of meningitis only model*.

##### *Proof*

To obtain an invariant region that shows that the solution is bounded, we have

$${N}_{1}=S+{I}_{m}+ {V}_{m}+R$$

$$\frac{d{N}_{1}}{dt}=\lambda -\mu N-{\delta }_{1}{I}_{m} \Rightarrow \frac{d{N}_{1}}{dt}\le \lambda -\mu {N}_{1}$$

Thus $$0\le {N}_{1}\le \frac{\lambda }{\mu }$$

There for the dynamical system is bounded.

#### Existence of the disease-free equilibrium point of the meningitis only model

The disease-free equilibrium point of the system is obtained by making all the equations equal to zero, providing that $\{{I}_{m}=0\}$. There for the disease free equilibrium point is

$${{E}^{O}}_{m}=\left({S}^{O},{{V}_{m }}^{O},{{I}_{m }}^{O} {,R}^{O} \right)=\left(\frac{\lambda }{\mu }\left[\frac{\left(1-\pi \right)\left(\mu +\phi \right)+\uppi \phi }{\left(\upmu +\phi \right)}\right], \frac{\mathrm{\pi \lambda }}{\left(\upmu +\phi \right)},\mathrm{0,0}\right)$$

#### Effective reproduction number of meningitis only model

The reproduction number is the expected number of secondary cases produced by one typical infectious inter in a completely susceptible population during its infectious period. We manipulated the effective reproduction number by using the next generation matrix method on the system and obtained ${R}_{eff(m)}={\alpha }_{2}\frac{\lambda }{\mu }\left(\frac{\left(1-\pi \right)\left(\mu +\phi \right)+\uppi \phi }{\left(\upmu +\phi \right)\left({\tau }_{1}+{\delta }_{1}+\mu \right)}\right)$. The basic reproduction number, which manipulated in the absence of an intervention, is given by ${R}_{om}=\frac{{ \lambda \alpha }_{2}}{\mu \left({\tau }_{1}+{\delta }_{1}+\mu \right)}$.

#### Local stability of the disease-free equilibrium point of the meningitis only model

##### **Theorem 3**

*The disease-free equilibrium point* ${{E}^{O}}_{m}=\left(\frac{\lambda }{\mu }\left[\frac{\left(1-\pi \right)\left(\mu +\phi \right)+\uppi \phi }{\left(\upmu +\phi \right)}\right], \frac{\mathrm{\pi \lambda }}{\left(\upmu +\phi \right)},\mathrm{0,0}\right)$ *of the model in system* (2) *is locally asymptotically stable if the effective reproduction number* ${R}_{eff(m)}<1$ and *unstable if* ${R}_{eff(m)}>1$.

##### *Proof*

The Jacobean matrix $J\left({{E}^{O}}_{m}\right)$ of the model (2) with respect to $\left(S ,{V}_{m, } {I}_{m } , R\right)$ at the disease-free equilibrium point ${{E}^{O}}_{m}=\left(\frac{\lambda }{\mu }\left[\frac{\left(1-\pi \right)\left(\mu +\phi \right)+\uppi \phi }{\left(\upmu +\phi \right)}\right], \frac{\mathrm{\pi \lambda }}{\left(\upmu +\phi \right)},\mathrm{0,0}\right)$ is follows.

$$J\left({{E}^{O}}_{m} \right)=\left[\begin{array}{cccc}-\mu & \phi & -{ \alpha }_{2}\frac{\lambda }{\mu }\left[\frac{\left(1-\pi \right)\left(\mu +\phi \right)+\uppi \phi }{\left(\upmu +\phi \right)}\right]& \rho \\ 0& -\left(\upmu +\phi \right)& 0& 0\\ 0& 0& { \alpha }_{2}\frac{\lambda }{\mu }\left[\frac{\left(1-\pi \right)\left(\mu +\phi \right)+\uppi \phi }{\left(\upmu +\phi \right)}\right]-\left( {\tau }_{1}+{\delta }_{1}+\mu \right)& 0\\ 0& 0& {\tau }_{1}& -\left(\rho +\mu \right)\end{array}\right]$$

For simplicity let ${h}_{1}=\frac{\lambda }{\mu }\left[\frac{\left(1-\pi \right)\left(\mu +\phi \right)+\uppi \phi }{\left(\upmu +\phi \right)}\right] \; and \; { h}_{2}=\left( {\tau }_{1}+{\delta }_{1}+\mu \right)$

Then, the corresponding characteristic equation is

$$\Rightarrow \left(-\mu -{\bigwedge }_{1}\right)\left(-\left(\mu +\phi \right)-{\bigwedge }_{2}\right)\left(\left({ \alpha }_{2}{h}_{1}-{h}_{2}\right)-{\bigwedge }_{3}\right)\left(-\left(\rho +\mu \right)-{\bigwedge }_{4}\right)=0.$$

$\Rightarrow { \bigwedge }_{1}=-\mu$ or ${\bigwedge }_{2}=-\left(\mu +\phi \right)$ or ${\bigwedge }_{3} =-\left({ \alpha }_{2}{h}_{1}-{h}_{2}\right)$ or ${ \bigwedge }_{4}=-\left(\rho +\mu \right)$

Hence, all the roots are negative, and ${\bigwedge }_{3}$ can be written as follow after rearranging the term using the above assumption. ${\bigwedge }_{3}= \left({\tau }_{1}+{\delta }_{1}+\mu \right)\left( {R}_{eff(m)}-1\right)$.

The disease-free equilibrium point is locally asymptotically stable if and only if ${R}_{eff(m)} <1$; otherwise, it is unstable if ${R}_{eff(m)}>1$.

Biologically, these results indicate that meningitis disease can be dies out from the communities when ${R}_{eff(m)}<1$, providing that the initial size of the subpulation of the submodel are in the region of attraction of ${{E}^{O}}_{m}$.

#### Global stability of the disease-free equilibrium point of the meningitis only model

##### **Theorem 4**

*The disease-free equilibrium is globally asymptotically stable if* ${R}_{eff(m)}< 1$.

##### *Proof*

To prove the global asymptotic stability (G A S) of the disease-free equilibrium point, we used the Lyapunov function method. We defined a Lyapunov function ${L}_{1}$ such that;

${L}_{1}=a{I}_{m } \; where \; a=\frac{1}{{\tau }_{1}+{\delta }_{1}+\mu }$ ⇒ $\frac{d{L}_{1}}{dt}=\frac{{f}_{1}S}{{\tau }_{1}+{\delta }_{1}+\mu }-{I}_{m}$. However, we do have ${f}_{1} ={ \alpha }_{2}{I}_{m} \; {\mathrm{ and }} \; {N}_{1}=S+{V}_{m}=\frac{\lambda }{\mu }$ ⇒ $\frac{d{L}_{1}}{dt}\le \left[{R}_{eff(m)}-1\right]{I}_{m}$ so $\frac{d{L}_{1}}{dt}< 0\, if \,{R}_{eff\left(m\right)}< 1$ and furthermore, $\frac{dL}{dt}=0$ if ${I}_{m} =0\, or\, {R}_{eff(m)} = 1$, holding these we can see that; $\left(\frac{\lambda }{\mu }\left[\frac{\left(1-\pi \right)\left(\mu +\phi \right)+\uppi \phi }{\left(\upmu +\phi \right)}\right], \frac{\mathrm{\pi \lambda }}{\left(\upmu +\phi \right)},\mathrm{0,0}\right)$ is the only singleton set in $\{\left(S, {V}_{m},{I}_{m},R\right)\in {\Omega }_{1}:\frac{d{L}_{1}}{dt}=0\}$. Therefore, by the principle of (LaSalle, 1976), DFE is globally asymptotically stable if ${R}_{eff\left(m\right)}< 1$.

#### Existence of the endemic equilibrium point of the meningitis-only model

The endemic equilibrium point of the model is denoted by ${E}_{m}^{*}=\left({S}^{*},{{I}_{m}}^{*}, {{V}_{m, }}^{*}{,R}^{*}\right)$, which occurs when the disease persists among the community and obtained by making the system equal to zero.

The endemic equilibrium point of the system is ${E}_{m}^{*}=\left({S}^{*},{{I}_{m }}^{*} , {{V}_{m }}^{*}{,R}^{*}\right)$

$${S}^{*}=\frac{\left(1-\pi \right)\lambda \left(\rho +\mu \right)\left( {\tau }_{1}+{\delta }_{1}+\mu \right)\left(\upmu +\phi \right)+\phi \mathrm{\Pi \lambda }\left(\rho +\mu \right)\left( {\tau }_{1}+{\delta }_{1}+\mu \right)}{\left(\rho +\mu \right)\left( {\tau }_{1}+{\delta }_{1}+\mu \right)\left(\upmu +\phi \right)\left({f}_{1}+\mu \right)-\left(\upmu +\phi \right)\rho {\tau }_{1}{f}_{1}}$$

$${{I}_{m }}^{*}=\left(\frac{{f}_{1}}{\left( {\tau }_{1}+{\delta }_{1}+\mu \right)}\right)\frac{\left(1-\pi \right)\lambda y+\phi \mathrm{\Pi \lambda }\left(\rho +\mu \right)\left( {\tau }_{1}+{\delta }_{1}+\mu \right)}{y\left({f}_{1}+\mu \right)-\left(\upmu +\phi \right)\rho {\tau }_{1}{f}_{1}}$$

$${{V}_{mp }}^{*}=\frac{\mathrm{\Pi \lambda }}{\left(\upmu +\phi \right)}$$

$${R}^{*}=\left(\frac{{\tau }_{1}{f}_{1}}{\left(\rho +\mu \right)\left( {\tau }_{1}+{\delta }_{1}+\mu \right)}\right)\frac{\left(1-\pi \right)\lambda y+\phi \mathrm{\Pi \lambda }\left(\rho +\mu \right)\left( {\tau }_{1}+{\delta }_{1}+\mu \right)}{y\left({f}_{1}+\mu \right)-\left(\upmu +\phi \right)\rho {\tau }_{1}{f}_{1}}\;\;where \; y=\left(\rho +\mu \right)\left( {\tau }_{1}+{\delta }_{1}+\mu \right)\left(\upmu +\phi \right)$$

$\Rightarrow {f}_{1}=\left(\frac{\left({\mu }^{3}+{\mu }^{2}\rho +{\mu }^{2}{\delta }_{1}+\mu \rho {\delta }_{1}+{\mu }^{2}{\tau }_{1}+\mu \rho {\tau }_{1}\right)}{\left({\mu }^{2}+\mu \rho +\mu {\delta }_{1}+\rho {\delta }_{1}+\mu {\tau }_{1}\right)}\right)\left[{R}_{eff(m)}-1\right]$. Thus, ${f}_{1}>0$ if and only if ${R}_{eff(m)}>1$ and the system has unique endemic equilibrium point.

#### Local stability of the endemic equilibrium point of the meningitis-only model

##### **Theorem 5**

*The endemic equilibrium point of system* (2), ${E}_{m}^{*}=\left({S}^{*},{{I}_{m }}^{*} , {{V}_{m }}^{*}{,R}^{*}\right)$ *is locally asymptotically stable for the reproduction number* ${R}_{eff(m)} >1$.

##### *Proof*

To show the local stability of the endemic equilibrium point, we use the Jacobian matrix and Routh Hurwitz stability criteria.

The Jacobian matrix of the dynamical system at the endemic equilibrium point is

$$\mathrm{J}({E}_{m}^{*})=\left[\begin{array}{cccc}-\left({ \alpha }_{2}{{I}_{m }}^{*} +\mu \right)& -{ \alpha }_{2}{S}^{*}& \phi & \rho \\ { \alpha }_{2}{{I}_{m }}^{*}& { \alpha }_{2}{S}^{*}-\left( {\tau }_{1}+{\delta }_{1}+\mu \right)& 0& 0\\ 0& 0& -\left(\upmu +\phi \right)& 0\\ 0& {\tau }_{1}& 0& -\left(\rho +\mu \right)\end{array}\right]$$

Then, the characteristic equation is given by

$$\left({B}_{1} -\lambda \right)\left|\begin{array}{ccc}{B}_{5}-\lambda & 0& 0\\ 0& {B}_{3}-\lambda & 0\\ {\tau }_{1}& 0& {B}_{4}-\lambda \end{array}\right|+{B}_{2}\left|\begin{array}{ccc}{B}_{2}& 0& 0\\ 0& {B}_{3}-\lambda & 0\\ 0& 0& {B}_{4}-\lambda \end{array}\right|+\phi \left|\begin{array}{ccc}{B}_{2}& {B}_{5}-\lambda & 0\\ 0& 0& 0\\ 0& {\tau }_{1}& {B}_{4}-\lambda \end{array}\right|-\rho \left|\begin{array}{ccc}{B}_{2}& {B}_{5}-\lambda & 0\\ 0& 0& {B}_{3}-\lambda \\ 0& {\tau }_{1}& 0\end{array}\right|=0$$

where $${B}_{1}= -\left({ \alpha }_{2}{S}^{*} +\mu \right), {B}_{2}={ \alpha }_{2}{S}^{*}, {B}_{3}=-\left(\upmu +\phi \right), {B}_{4}=-\left(\rho +\mu \right) \; and\; {B}_{5}=\left[{B}_{2}-\left( {\tau }_{1}+{\delta }_{1}+\mu \right)\right]$$

$$\Rightarrow {a}_{0}{\lambda }^{4}+{a}_{1}{\lambda }^{3}+{a}_{2}{\lambda }^{2}+{a}_{3}\lambda +{a}_{4}=0$$

where $${a}_{0}=1, {a}_{1}=-\left({B}_{1}+{B}_{3}+{B}_{4}+{B}_{5}\right), {a}_{2}=\left[{B}_{1}{B}_{3}+{{B}_{2}}^{2}+{B}_{4}{B}_{5}+{B}_{1}{B}_{4}+{B}_{3}{B}_{4}+{B}_{1}{B}_{5}+{B}_{3}{B}_{5}\right], {a}_{3}=-\left({B}_{1}{B}_{3}{B}_{4}+{B}_{4}{{B}_{2}}^{2}+{\tau }_{1}\rho {B}_{2}+{{{B}_{2}}^{2}B}_{3}+{B}_{1}{B}_{3}{B}_{5}+{B}_{1}{B}_{4}{B}_{5}+{B}_{3}{B}_{4}{B}_{5}\right) \; and \; {a}_{4}={B}_{1}{B}_{3}{B}_{4}{B}_{5}+{{{B}_{2}}^{2}B}_{3}{B}_{4}+{\tau }_{1}\rho {B}_{2}{B}_{3}$$

Here, we have to check the necessary condition of the Routh-Hurwitz stability criteria. Since ${a}_{0}=1$ is positive in sign, all ${a}_{1}$, ${a}_{2}, \; {a}_{3}$ and ${a}_{4}$ should be positives and hence ${a}_{1}=-\left({B}_{1}+{B}_{3}+{B}_{4}+{B}_{5}\right)$, but ${B}_{1}<0$, ${B}_{2}>0$, ${B}_{3}<0, \; {B}_{4}<0 \; and \; { B}_{5}<0$.

We know that the sum of negative numbers is always negative, $-\left({B}_{1}+{B}_{3}+{B}_{4}+{B}_{5}\right)$ is positive. Therefore, ${a}_{1}$ is positive in sign.

Following the same procedures ${a}_{2}>0$, ${a}_{3}>0$ and ${a}_{4}>0$. From our algebraic manipulation done above, all the coefficients of the characteristic’s polynomial are positive whenever ${\mathcal{R}}_{eff(m)}>1$. Then, we can apply the Routh-Hurwitz criteria to determine the sign of the roots of the characteristic equation without calculating the values of the roots of the characteristics equation ${{a}_{0}\lambda }^{4}+ {a}_{1}{\lambda }^{3}+{a}_{2}{\lambda }^{2}+{a}_{3}\lambda +{a}_{4}=0$

 where $${b}_{1}=\frac{-1}{{a}_{1}}\left|\begin{array}{cc}{a}_{0}& {a}_{2}\\ {a}_{1}& {a}_{3}\end{array}\right|=\frac{-1}{{a}_{1}}\left({a}_{0}{a}_{3}-{a}_{1}{a}_{2}\right) \; \; and \; \; {b}_{1}=\frac{-1}{{a}_{1}}\left({a}_{3}-{a}_{1}{a}_{2}\right)$$

$\Rightarrow {b}_{1}>0$ $\text{for } \; {R}_{eff(m)} >1$. In the same procedure, ${b}_{2}>0$, ${c}_{1}>0$, ${d}_{1}>0$.

Hence, the first column of the Routh Hurwitz array has no sign change, and then the root of the characteristic equation of the dynamical system is negative. Therefore, the endemic equilibrium point of the dynamical system is locally asymptotically stable.

#### Global stability of the endemic equilibrium point of the meningitis only model

##### **Theorem 6**

*If* ${R}_{eff\left(m\right)}>1$, *the endemic equilibrium of the model* (2) *is globally asymptotically stable*.

##### *Proof*

Systematically, we define an appropriate Lyapunov function ${L}_{2}$ such that;

$${L}_{2}=\left(S-{S}^{*}+{S}^{*}ln\frac{{S}^{*}}{S}\right)+\left({I}_{m}-{{I}_{m}}^{*}+{{I}_{m}}^{*}ln\frac{{{I}_{m}}^{*}}{{I}_{m}}\right)+\left({V}_{m}-{{V}_{m}}^{*}+{{V}_{m}}^{*}ln\frac{{{V}_{m}}^{*}}{{V}_{m}}\right)+\left(R-{R}^{*}+{R}^{*}ln\frac{{R}^{*}}{R}\right)$$

Then, after differentiating ${L}_{2}$ with respect to time, $t$ we have the following.

$$\frac{{dL}_{2}}{dt}=\left(\frac{S-{S}^{*}}{S}\right)\frac{dS}{dt}+\left(\frac{{I}_{m}-{{I}_{m}}^{*}}{{I}_{m}}\right)\frac{d{I}_{m}}{dt}+\left(\frac{{{{V}_{m}-V}_{m }}^{*}}{{V}_{m}}\right)\frac{d{V}_{m}}{dt}+\left(\frac{R-{R}^{*}}{R}\right)\frac{dR}{dt}$$

Then, substituting $$\frac{dS}{dt}, \frac{d{I}_{m}}{dt}, \frac{d{V}_{m}}{dt}, \frac{dR}{dt}$$

$\Rightarrow \frac{{dL}_{2}}{dt}={Q}_{1}-{Q}_{2}$, where ${Q}_{1}=\lambda +{{I}_{m}}^{*}{\tau }_{1}+{{I}_{m}}^{*}{\delta }_{1}+{{I}_{m}}^{*}\mu +{{V}_{m }}^{*}\mu +{{V}_{m }}^{*}\phi +{R}^{*}\rho +{R}^{*}\mu +{{S}^{*}f}_{1}+{S}^{*}\mu \; and \; {Q}_{2}=S\mu +{I}_{m}{\delta }_{1}+{I}_{m}\mu +{V}_{m}\mu +R\mu +\left(\frac{{S}^{*}\left[\left(1 -\pi \right)\lambda +\rho R+\phi {V}_{m} \right]}{S}\right)+\left(\frac{{{I}_{m}}^{*}\left[{f}_{1}S\right]}{{I}_{m}}\right)+\left(\frac{{{V}_{m }}^{*}\left[\pi \lambda \right]}{{V}_{m}}\right)+\left(\frac{{R}^{*}\left[{\tau }_{1}{I}_{m}\right]}{R}\right)$. Thus, if ${Q}_{1} <{Q}_{2},$ then $\frac{{dL}_{2}}{dt}\le 0$, and $\frac{{dL}_{2}}{dt}=0$ if and only if $S={S}^{*}, {I}_{m}={{I}_{m}}^{*}, { V}_{m}={{V}_{m }}^{*} \; and \; R={R}^{*} .$

From this, we see that ${{E}^{*}}_{m}=\left({S}^{*}, {{I}_{m}}^{*}, {{V}_{m }}^{*}{, R}^{*}\right)$ is the largest compact invariant singleton set in $\left\{\left({S}^{*}, {{I}_{m}}^{*}, {{V}_{m }}^{*}{, R}^{*}\right)\epsilon { \Omega }_{1}: \frac{{dL}_{2}}{dt}=0\right\}$.

Therefore, by the principle of (LaSalle, 1976), the endemic equilibrium $({{E}^{*}}_{m})$ is globally asymptotically stable in the invariant region if ${Q}_{1} <{Q}_{2}$.

### Pneumonia only model

Pneumonia only model is obtained from the full meningitis and pneumonia co infectious model (1) by setting ${ I}_{m}={I}_{mp}={T}_{mp}=0$. After such algebraic operations, we do have the following of dynamical system.

3 $$\left.\begin{array}{c}\frac{dS}{dt}=\left(1-\pi \right)\lambda +\rho R+\phi {V}_{p} -\left({f}_{2} +\mu \right)S\\ \frac{d{I}_{P}}{dt}={f}_{2}S-\left({\tau }_{3} +{\delta }_{2} +\mu \right){I}_{p} \\ \frac{d{V}_{p}}{dt}=\pi \lambda -\left(\upmu +\phi \right){V}_{p} \\ \frac{d\mathrm{R}}{dt}={\tau }_{3}{I}_{P}-\left(\rho +\mu \right)R \end{array} \right\}$$

#### Positivity of solutions of the pneumonia only model

To be the above dynamical systems (3) is epidemiologically meaningful and posed; we need to prove that all the state variables of dynamical systems are non-negative.

##### **Theorem 7**

*All the populations of the system with positive initial conditions are nonnegative*.

##### *Proof*

Assume $S\left(0\right)>0$, ${I}_{p}\left(0\right)>0$, ${V}_{p}\left(0\right)>0 \; and \; R\left(0\right)>0$ are positive for time $t>0$ and for all nonnegative parameters. Let us take $T=\mathrm{sup}\{t>0$ such that $S\left({t}^{{\prime}}\right)>0 , {I}_{p}\left({t}^{{\prime}}\right)>0, {V}_{p}\left({t}^{{\prime}}\right)>0$ and $R\left({t}^{{\prime}}\right)>0 , {t}^{{\prime}}\in [0, t] \}$.

From the initial condition, all the state variables are non-negative at the initial time and $T>0$.

Moreover, the first equation of the given system $\frac{dS}{dt}=\left(1-\pi \right)\lambda +\rho R+\phi {V}_{p} -\left({f}_{2} +\mu \right)S$ can be integrated using integrating factor $IF={e}^{{\int }_{0}^{T}\left({f}_{2} +\mu \right)d{t}^{{\prime}}}$

$$S\left(t\right)={k}_{5}S\left(0\right)+{k}_{5}\left[{\int }_{0}^{T}{e}^{{\int }_{0}^{T}\left({f}_{2} +\mu \right)d{t}^{{\prime}}}[\left(1-\pi \right)\lambda +\rho R+\phi {V}_{p}]dt\right]>0$$

where ${k}_{5}={e}^{-{\int }_{0}^{T}\left({f}_{2} +\mu \right)d{t}^{{\prime}}}$ which is nonnegative and exponential function.

Which implies that $S\left(t\right)>0$. Following the same procedures, we have ${I}_{p}\left(t\right)>0$, ${V}_{p}\left(t\right)>0$ and $R\left(t\right)>0$. Therefore, from above proof, we can conclude that whenever the initial values of the systems are all nonnegative, then all the solutions of our dynamical system are positive.

#### Invariant region of the pneumonia only model

##### **Theorem 8**

*The dynamical system* (3) *is positively invariant in the closed invariant set* ${\Omega }_{2} =\{\left(S, {I}_{p}, {V}_{p, } R\right)\epsilon {\mathbb{R}}_{+}^{4}:{N}_{2}\le \frac{\lambda }{\mu }\}$ where the total human population of the system is assumed to be ${N}_{2}$.

##### *Proof*

To determine an invariant region that elucidate the boundedness of solution, we have

$${N}_{2}=S+ {I}_{p}+{V}_{p}+R$$ ⇒ $$\frac{d{N}_{2}}{dt}=\lambda -\mu {N}_{2}-{\delta }_{2}{I}_{p}$$

⇒ $\frac{d{N}_{2}}{dt}\le \lambda -\mu {N}_{2}$ ⇒$\frac{d{N}_{2}}{dt}+\mu {N}_{2}\le \lambda$, then using an integrating factor of $IF={e}^{\int \mu dt}={e}^{\mu t}$

$$\Rightarrow \underset{t\to \infty }{\mathrm{lim}}{N}_{2}\left(t\right)\le \frac{\lambda }{\mu }$$ Thus $$0\le {N}_{2}\left(t\right)\le \frac{\lambda }{\mu }$$

Therefore, the dynamical system is bounded.

#### Existence of the disease-free equilibrium point of the pneumonia-only model

The disease-free equilibrium point is obtained by making all the equations of the system equal to zero, providing that $\{{I}_{p}=0\}$. Thus, the disease-free equilibrium point of the system is given by

$${{E}^{O}}_{p}=\left({S}^{O},{{V}_{p, }}^{O} {{I}_{p}}^{O}{,R}^{O} \right)=\left(\frac{\lambda }{\mu }\left[\frac{\left(1-\pi \right)\left(\mu +\phi \right)+\uppi \phi }{\left(\upmu +\phi \right)}\right], \frac{\mathrm{\pi \lambda }}{\left(\upmu +\phi \right)},\mathrm{0,0}\right)$$

#### Effective reproduction number of pneumonia only model

The reproduction number is the expected number of secondary cases produced by one typical infectious inter in a completely susceptible population during its infectious period.

Therefore, the effective reproduction number of pneumonia-infected only model is ${R}_{eff(p)}=\frac{\lambda }{\mu }\left(\frac{\left(1-\pi \right)\left(\mu +\phi \right)+\uppi \phi }{\left(\upmu +\phi \right){(\tau }_{3} +{\delta }_{2} +\mu )}\right)$ and the basic reproduction number determined in the absence of an intervention is given by ${R}_{op}=\frac{{ \lambda \alpha }_{1}}{({\tau }_{3} +{\delta }_{2} +\mu )\mu }$.

#### Local stability of the disease-free equilibrium point of the pneumonia-only model

##### **Theorem 9**

*The disease-free equilibrium point* ${{E}^{O}}_{p}=\left(\frac{\lambda }{\mu }\left[\frac{\left(1-\pi \right)\left(\mu +\phi \right)+\uppi \phi }{\left(\upmu +\phi \right)}\right], \frac{\mathrm{\pi \lambda }}{\left(\upmu +\phi \right)},\mathrm{0,0}\right)$ *of the model in system* (3) *is locally asymptotically stable if the effective reproduction number* ${R}_{eff(p)}<1$ *and is unstable if* ${R}_{eff(p)}>1$.

##### *Proof*

The Jacobean matrix $J\left({{E}^{O}}_{p}\right)$ of system (3) with respect to $\left(S ,{V}_{p, } {I}_{p}, R\right)$ at the disease-free equilibrium point is,

$$J\left({{E}^{O}}_{p} \right)=\left[\begin{array}{cccc}-\mu & \phi & -{ \alpha }_{1}\frac{\lambda }{\mu }\left[\frac{\left(1-\pi \right)\left(\mu +\phi \right)+\uppi \phi }{\left(\upmu +\phi \right)}\right]& \rho \\ 0& -\left(\upmu +\phi \right)& 0& 0\\ 0& 0& { \alpha }_{1}\frac{\lambda }{\mu }\left[\frac{\left(1-\pi \right)\left(\mu +\phi \right)+\uppi \phi }{\left(\upmu +\phi \right)}\right]-\left( {\tau }_{3} +{\delta }_{2} +\mu \right)& 0\\ 0& 0& {\tau }_{3}& -\left(\rho +\mu \right)\end{array}\right]$$

For simplicity let ${h}_{1}=\frac{\lambda }{\mu }\left[\frac{\left(1-\pi \right)\left(\mu +\phi \right)+\uppi \phi }{\left(\upmu +\phi \right)}\right] \; and \; { h}_{3}=\left( {\tau }_{3} +{\delta }_{2} +\mu \right)$

Then, the corresponding characteristic equation is

$$\left(-\mu -{\bigwedge }_{1}\right)\left(-\left(\upmu +\phi \right)-{\bigwedge }_{2}\right)\left(\left({ \alpha }_{1}{h}_{1}-{h}_{3}\right)-{\bigwedge }_{3}\right)\left(-\left(\rho +\mu \right)-{\bigwedge }_{4}\right)=0$$

$$\Rightarrow { \bigwedge }_{1}=-\mu \; or \; {\bigwedge }_{2}=-\left(\mu +\phi \right) \; or \; {\bigwedge }_{3} =-\left({ \alpha }_{1}{h}_{1}-{h}_{3}\right) \; or \; {\bigwedge }_{4}=-\left(\rho +\mu \right)$$

Hence, all the roots are negative and ${\bigwedge }_{3}$ can be written as follow after rearranging the term using the above assumption ${\bigwedge }_{3}= \left({\tau }_{3} +{\delta }_{2} +\mu \right)\left( {R}_{eff(p)}-1\right)$.

Therefore, the disease-free equilibrium point is locally asymptotically stable if and only if ${R}_{eff(p)}<1$; otherwise, it is unstable, if ${R}_{eff(p)}>1$.

Biologically, these results indicate that pneumonia disease can be dies out from the communities when ${R}_{eff(p)}<1$, providing that the initial size of the subpulation of the submodel are in the region of attraction of ${{E}^{O}}_{p}$.

#### Global stability of the disease-free equilibrium point of the pneumonia-only model

##### **Theorem 10**

*The disease-free equilibrium is globally asymptotically stable if* ${R}_{eff(P)}< 1$.

##### *Proof*

To prove the global asymptotic stability (G A S) of the disease-free equilibrium point, we use the method of Lyapunov functions.

We defined a Lyapunov function ${L}_{2}$ such that ${L}_{2}=b{I}_{p }$ where $b=\frac{1}{{\tau }_{3} +{\delta }_{2} +\mu }$

Then, differentiating ${L}_{2}$ with respect to $t$ $\frac{d{L}_{2}}{dt}=\frac{{f}_{2}S}{\left( {\tau }_{3} +{\delta }_{2} +\mu \right)}-{I}_{P}$

 ⇒ $\frac{d{L}_{2}}{dt}\le \left[{R}_{eff(p)}-1\right]{I}_{p}$ so, $\frac{d{L}_{2}}{dt} \le 0$ if ${R}_{eff\left(p\right)} \le 1$ and furthermore, $\frac{d{L}_{2}}{dt}=0$ if $\mathrm{p} = 0$ or ${R}_{eff(p)} = 1$. Holding these, we can see that $\left(\frac{\lambda }{\mu }\left[\frac{\left(1-\pi \right)\left(\mu +\phi \right)+\uppi \phi }{\left(\upmu +\phi \right)}\right], \frac{\mathrm{\pi \lambda }}{\left(\upmu +\phi \right)},\mathrm{0,0}\right)$ is the only singleton in $\{\left(S, {V}_{p},{I}_{p},R\right)\in {\Omega }_{2}:\frac{d{L}_{2}}{dt}=0\}$. Therefore, by the principle of (LaSalle, 1976), DFE is globally asymptotically stable if ${R}_{eff\left(p\right)}< 1$.

#### Existence of the endemic equilibrium point of the pneumonia-only model

To determine conditions for the existence of an arbitrary equilibrium point(s) for pneumonia infection in the population, the equations of the model of pneumonia infected only model are needed to be solved in terms of the force of infection ${f}_{2}={\alpha }_{1}{{I}_{P}}^{*}$ at the equilibrium point.

The endemic equilibrium point of the model is denoted by ${E}_{p}^{*}=\left({S}^{*}, {{I}_{p}}^{*}, {{V}_{p, }}^{*}{,R}^{*}\right)$, which occurs when the disease persists among the community and is manipulated by making the equations in corresponding dynamical system equal to zero. Thus, we get

$${S}^{*}=\frac{\left(\rho +\mu \right)\left({\tau }_{3} +{\delta }_{2} +\mu \right)\left(1-\pi \right)\lambda \left(\upmu +\phi \right)+\phi \mathrm{\Pi \lambda }\left(\rho +\mu \right)\left({\tau }_{3} +{\delta }_{2} +\mu \right)\left(\upmu +\phi \right)}{\left({f}_{2} +\mu \right)\left(\rho +\mu \right)\left({\tau }_{3} +{\delta }_{2} +\mu \right)\left(\upmu +\phi \right)-{\left(\upmu +\phi \right)\rho \tau }_{3}{f}_{2}}$$

$${{I}_{p}}^{*}=\frac{{f}_{2}{S}^{*}}{\left({\tau }_{3} +{\delta }_{2} +\mu \right)}$$

$${{V}_{p }}^{*}=\frac{\mathrm{\Pi \lambda }}{\left(\upmu +\phi \right)}$$

$${R}^{*}=\frac{{\tau }_{3}{f}_{2}{S}^{*}}{\left(\rho +\mu \right)\left({\tau }_{3} +{\delta }_{2} +\mu \right)}$$

Now substituting ${S}^{*} \; in \; to \; {{I}_{p}}^{*}$ we do have ${f}_{2}=\frac{\left(\rho +\mu \right)\left({\tau }_{3} +{\delta }_{2} +\mu \right)\mu }{\left(\left(\rho +\mu \right)\left({\tau }_{3} +{\delta }_{2} +\mu \right)-{\rho \tau }_{3}\right)}\left({R}_{eff(p)}-1\right)$

Thus, ${f}_{2}=\frac{\left(\rho +\mu \right)\left({\tau }_{3} +{\delta }_{2} +\mu \right)\mu }{\left(\left(\rho +\mu \right)\left({\tau }_{3} +{\delta }_{2} +\mu \right)-{\rho \tau }_{3}\right)}\left({R}_{eff(p)}-1\right)>0$ if and only if ${R}_{eff(p)}>1$ and obviously $\left(\rho +\mu \right)\left({\tau }_{3} +{\delta }_{2} +\mu \right)-{\rho \tau }_{3}>0$ Therefore, the system has unique endemic equilibrium point if ${R}_{eff(p)}>1$

#### Local stability of the endemic equilibrium point of the pneumonia-only model

##### **Theorem 11**

*The endemic equilibrium point* ${E}_{p}^{*}=\left({S}^{*}, {{I}_{p}}^{*}, {{V}_{p, }}^{*}{,R}^{*}\right)$ *is locally asymptotically stable if the reproduction number* ${R}_{eff(p)} >1$.

##### *Proof*

To show the local stability of the endemic equilibrium point, we use the Jacobian matrix and Routh Hurwitz stability criteria. Then, the Jacobian matrix of the dynamical system at the endemic equilibrium point is

$$J\left({{E}_{p}}^{*}\right)=\left[\begin{array}{cccc}-\left({{{\alpha }_{1}I}_{p}}^{*} +\mu \right)& -{ \alpha }_{1}{S}^{*}& \phi & \rho \\ {{{\alpha }_{1}I}_{p}}^{*}& { \alpha }_{1}{S}^{*}-\left( {\tau }_{3} +{\delta }_{2} +\mu \right)& 0& 0\\ 0& 0& -\left(\upmu +\phi \right)& 0\\ 0& {\tau }_{3}& 0& -\left(\rho +\mu \right)\end{array}\right]$$

Thus, the characteristic equation of the above Jacobian matrix is given by

$$\left|\begin{array}{cccc}{D}_{1}-\lambda & { D}_{2}& \phi & \rho \\ {D}_{3}& {D}_{5}-\lambda & 0& 0\\ 0& 0& {D}_{6}-\lambda & 0\\ 0& {\tau }_{3}& 0& {D}_{7}-\lambda \end{array}\right|=0$$, Where $${D}_{1}=-\left({{{\alpha }_{1}I}_{p}}^{*} +\mu \right)=-\left({\alpha }_{1}\frac{{f}_{2}{S}^{*}}{\left({\tau }_{3} +{\delta }_{2} +\mu \right)} +\mu \right)$$

$${D}_{2}=\frac{-{ \alpha }_{1}\left(\rho +\mu \right)\left({\tau }_{3} +{\delta }_{2} +\mu \right)\left(1-\pi \right)\lambda \left(\upmu +\phi \right)-{\alpha }_{1}\phi \mathrm{\Pi \lambda }\left(\rho +\mu \right)\left({\tau }_{3} +{\delta }_{2} +\mu \right)\left(\upmu +\phi \right)}{\left({f}_{2} +\mu \right)\left(\rho +\mu \right)\left({\tau }_{3} +{\delta }_{2} +\mu \right)\left(\upmu +\phi \right)-{\left(\upmu +\phi \right)\rho \tau }_{3}{f}_{2}}$$

$${D}_{3}={{{\alpha }_{1}I}_{p}}^{*}, \;\; {D}_{4}=\left( {\tau }_{3} +{\delta }_{2} +\mu \right), \;\; {D}_{5}={ -D}_{2}-{D}_{4}, \;\; {D}_{6}=-\left(\upmu +\phi \right) \; \; \text{and} \; {D}_{7}=-\left(\rho +\mu \right)$$

$${{a}_{0}\lambda }^{4}+{a}_{1}{\lambda }^{3}+{a}_{2}{\lambda }^{2}+{a}_{3}\lambda +{a}_{4}=0$$ where $${a}_{1}=-\left({D}_{1}+{D}_{5}+{D}_{6}+{D}_{7}\right), {a}_{2}=\left({D}_{1}{D}_{5}+{D}_{2}{D}_{3}+{D}_{1}{D}_{6}+{D}_{5}{D}_{6}+{D}_{1}{D}_{7}+{D}_{5}{D}_{7}+{D}_{6}{D}_{7}\right), {a}_{3}=-\left({D}_{1}{D}_{5}{D}_{6}+\left({{\tau }_{3}D}_{3}\right)\rho +{D}_{2}{D}_{3}{D}_{6}+{D}_{1}{D}_{5}{D}_{7}+{D}_{2}{D}_{7}{D}_{3}+{D}_{1}{D}_{6}{D}_{7}-{D}_{5}{D}_{6}{D}_{7}\right), {a}_{4}={D}_{1}{D}_{5}{D}_{6}{D}_{7}+{\left({{\tau }_{3}D}_{3}\right)\rho D}_{6}+{{D}_{2}{D}_{7}{D}_{3}D}_{6} , {a}_{0}=1.$$

Here, we have to check the necessary condition of the Routh-Hurwitz stability criteria since ${a}_{0}=1$ is positive in sign.

After some manipulation ${\mathrm{a}}_{1}=-\left({D}_{1}+{D}_{5}+{D}_{6}+{D}_{7}\right)>0$ for ${R}_{eff\left(p\right)}>1$.

$${a}_{2}=\left({D}_{1}{D}_{5}+{D}_{2}{D}_{3}+{D}_{1}{D}_{6}+{D}_{5}{D}_{6}+{D}_{1}{D}_{7}+{D}_{5}{D}_{7}+{D}_{6}{D}_{7}\right)>0$$ for $${R}_{eff(p)}>1$$.

In the same way for ${a}_{3} >0$ and ${a}_{4}>0$.

From the above algebraic manipulations, all the coefficients of the characteristic’s polynomial are positives whenever ${\mathcal{R}}_{eff(p)}>1$.

Then, we can apply the Routh-Hurwitz criteria to determine the sign of the roots of the characteristic equation without calculating the values of the root of the characteristics equation ${{a}_{0}\lambda }^{4}+ {a}_{1}{\lambda }^{3}+{a}_{2}{\lambda }^{2}+{a}_{3}\lambda +{a}_{4}=0$

 where $${b}_{1}=\frac{-1}{{a}_{1}}\left|\begin{array}{cc}{a}_{0}& {a}_{2}\\ {a}_{1}& {a}_{3}\end{array}\right|=\frac{-1}{{a}_{1}}\left({a}_{0}{a}_{3}-{a}_{1}{a}_{2}\right)$$

$${b}_{1}=\frac{-1}{{a}_{1}}\left({a}_{0}{a}_{3}-{a}_{1}{a}_{2}\right)$$ ⇒$${b}_{1}=\frac{-1}{{a}_{1}}\left({a}_{3}-{a}_{1}{a}_{2}\right)$$ ⇒$${b}_{1}>0$$

In the same procedure

$${b}_{2}=\frac{-1}{{a}_{1}}\left|\begin{array}{cc}{a}_{0}& {a}_{4}\\ {a}_{1}& 0\end{array}\right| \Rightarrow {b}_{2}=\frac{-1}{{a}_{1}}\left(-{{a}_{4}a}_{1}\right)=\frac{{{a}_{4}a}_{1}}{{a}_{1}}={a}_{4}>0$$⇒$${b}_{2}>0$$

Again $${c}_{1}=\frac{-1}{{b}_{1}}\left|\begin{array}{cc}{a}_{1}& {a}_{3}\\ {b}_{1}& {b}_{1}\end{array}\right|=\frac{-1}{{b}_{1}}\left({{b}_{1}a}_{1}-{{a}_{3}b}_{1}\right)=\left({a}_{3}-{a}_{1}\right) \Rightarrow {c}_{1}>0$$

Finally, $${d}_{1}=\frac{-1}{{c}_{1}}\left|\begin{array}{cc}{b}_{1}& {b}_{2}\\ {c}_{1}& 0\end{array}\right|=\frac{-1}{{c}_{1}}\left(-{c}_{1}{b}_{2}\right)={b}_{2}>0$$ $$\Rightarrow {d}_{1}>0$$

Hence, the first column of the Routh Hurwitz array has no sign change, and then the root of the characteristic equation of the dynamical system is negative. Hence, the endemic equilibrium point of the dynamical system is locally asymptotically stable.

#### Global stability of the endemic equilibrium point of the pneumonia-only model

##### **Theorem 12**

*If* ${R}_{eff\left(P\right)}>1$, *the endemic equilibrium of the model* (3) *is globally asymptotically stable*.

##### *Proof*

Systematically, we define an appropriate Lyapunov function ${L}_{4}$ such that;

$${L}_{4}=\left(S-{S}^{*}+{S}^{*}ln\frac{{S}^{*}}{S}\right)+\left({I}_{p}-{{I}_{p}}^{*}+{{I}_{p}}^{*}ln\frac{{{I}_{p}}^{*}}{{I}_{p}}\right)+\left({V}_{p}-{{V}_{p}}^{*}+{{V}_{p}}^{*}ln\frac{{{V}_{p}}^{*}}{{V}_{p}}\right)+\left(R-{R}^{*}+{R}^{*}ln\frac{{R}^{*}}{R}\right)$$

Then, after differentiating ${L}_{3}$ with respect to time $t$, we have the following.

$$\frac{{dL}_{4}}{dt}=\left(\frac{S-{S}^{*}}{S}\right)\frac{dS}{dt}+\left(\frac{{I}_{p}-{{I}_{p}}^{*}}{{I}_{p}}\right)\frac{d{I}_{p}}{dt}+\left(\frac{{{{V}_{p}-V}_{p}}^{*}}{{V}_{p}}\right)\frac{d{V}_{p}}{dt}+\left(\frac{R-{R}^{*}}{R}\right)\frac{dR}{dt}$$

Then, by substituting $\frac{dS}{dt}, \frac{d{I}_{p}}{dt}, \frac{d{V}_{p}}{dt}, \frac{dR}{dt}$ and simplifying

$\Rightarrow \frac{{dL}_{4}}{dt}={P}_{1}-{P}_{2}$ where ${P}_{1}=\lambda +{{S}^{*}f}_{2}+{S}^{*}\mu +{{I}_{p}}^{*}{\delta }_{2}+{{I}_{p}}^{*}\mu +{{I}_{p}}^{*}{\tau }_{3}+{{V}_{p }}^{*}\mu +{{V}_{p }}^{*}\phi +{R}^{*}\rho +{R}^{*}\mu$ and ${P}_{2}=\left(S\mu +{I}_{p}{\delta }_{2}+{I}_{p}\mu +{V}_{p}\mu +R\mu +\left(\frac{{S}^{*}\left[\left(1- \pi \right)\lambda +\rho R+\phi {V}_{p} \right]}{S}\right)+\left(\frac{{{I}_{p}}^{*}\left[{f}_{2}S\right]}{{I}_{p}}\right)+\left(\frac{{R}^{*}\left[{\tau }_{2}{I}_{p}\right]}{R}\right)+\left(\frac{{{V}_{p }}^{*}\left[\pi \lambda \right]}{{V}_{p}}\right)\right)$ Thus, if ${P}_{1} <{P}_{2},$ then $\frac{{dL}_{4}}{dt}\le 0$, and $\frac{{dL}_{3}}{dt}=0$ if and only if $S={S}^{*}, {I}_{p}={{I}_{p}}^{*}, { V}_{p}={{V}_{p }}^{*} \; and \; R={R}^{*} .$ from this, we see that ${{E}^{*}}_{p}=\left({S}^{*}, {{I}_{p}}^{*}, {{V}_{p }}^{*}{, R}^{*}\right)$ is the largest compact invariant singleton set in $\left\{\left({S}^{*}, {{I}_{p}}^{*}, {{V}_{p }}^{*}{, R}^{*}\right)\epsilon { \Omega }_{2}: \frac{{dL}_{4}}{dt}=0\right\}$

Therefore, by the principle of (LaSalle, 1976), the endemic equilibrium $({{E}^{*}}_{p})$ is globally asymptotically stable in the invariant region if ${P}_{1} <{P}_{2}$.

### Meningitis and pneumonia coinfection model

In this part, we have considered the dynamical system of meningitis and pneumonia coinfection model stated in Eq. (1).

#### Positivity of solutions of the model

In order to be the stated dynamical systems are epidemiologically meaningful and posed, it is needed to prove that all the state variables of dynamical systems are nonnegative.

##### **Theorem 13**

*All the populations of the system with positive initial conditions are nonnegative*.

*Assume* $S\left(0\right)>0 , {I}_{m}\left(0\right)>0, {I}_{p}\left(0\right)>0, {I}_{mp}\left(0\right)>0, {T}_{mP}\left(0\right)>0 , {V}_{mp}\left(0\right)>0$ *and* $R\left(0\right)>0$ *are positive for time* $t>0$ *for all non-negative parameters*.

##### *Proof*

First, let us take $T=\mathrm{sup}\{t>0$ such that $S\left({t}^{{\prime}}\right)>0 , {I}_{m}\left({t}^{{\prime}}\right)>0, {I}_{p}\left({t}^{{\prime}}\right)>0, {I}_{mp}\left({t}^{{\prime}}\right)>0, {T}_{mP}\left({t}^{{\prime}}\right)>0 , {V}_{mp}\left({t}^{{\prime}}\right)>0$ and $R\left({t}^{{\prime}}\right)>0 , {t}^{{\prime}}\in [0, t] \}$.

From the initial condition, all the state variables are non-negative at the initial time; then, $T>0$.

From first equation of system (1); $\frac{dS}{dt}+\left({f}_{1}+{f}_{2} +\mu \right)S=\left(1-\pi \right)\lambda +\rho R+\phi {V}_{mp}$ , Integrated by using the integrating factor $IF={e}^{{\int }_{0}^{T}\left({f}_{1}+{f}_{2} +\mu \right)d{t}^{{\prime}}}$

$$S\left(t\right)=S\left(0\right){e}^{-{\int }_{0}^{T}\left({f}_{1}+{f}_{2} +\mu \right)d{t}^{{\prime}}}+{e}^{-{\int }_{0}^{T}\left({f}_{1}+{f}_{2} +\mu \right)d{t}^{{\prime}}}\left[{\int }_{0}^{T}{e}^{{\int }_{0}^{T}\left({f}_{1}+{f}_{2} +\mu \right)d{t}^{{\prime}}}[\left(1-\pi \right)\lambda +\rho R+\phi {V}_{mp}]dt\right]$$

Let ${r}_{1}={e}^{-{\int }_{0}^{T}\left({f}_{1}+{f}_{2} +\mu \right)d{t}^{{\prime}}}$, which is nonnegative because the exponential function cannot be negative. Thus $S\left(t\right)={r}_{1}S\left(0\right)+{r}_{1}\left[{\int }_{0}^{T}{e}^{{\int }_{0}^{T}\left({f}_{1}+{f}_{2} +\mu \right)d{t}^{{\prime}}}[\left(1-\pi \right)\lambda +\rho R+\phi {V}_{mp}]dt\right]>0$

$\Rightarrow S\left(t\right)>0$, in the same procedure we have

$${I}_{p}\left(t\right)>0$$, $${I}_{m}\left(t\right)>0$$ , $${I}_{mp}\left(t\right)>0$$ , $${\mathrm{T}}_{mp}\left(t\right)>0$$, $${V}_{mp}\left(t\right)>0$$ and $$R\left(t\right)>0$$.

Therefore, from the above proof, we can conclude that whenever the initial values of the systems are all nonnegative, then all the solutions of our dynamical system are positive.

#### Invariant region

##### **Theorem 14**

*The dynamic system* (1) *is positively invariant in the closed invariant set*

$$\Omega =\left\{\left(S, {I}_{p}, {I}_{m, }{I}_{mp}, {T}_{mp}, {V}_{mp, }R\right)\epsilon {\mathbb{R}}_{+}^{7}:N\le \frac{\lambda }{\mu }\right\}.$$

##### *Proof*

To obtain an invariant region that shows that the solution is bounded, we have

$$N=S+ {I}_{p}+ {I}_{m}+{I}_{mp}+ {T}_{mp}+ {V}_{mp}+R \Rightarrow \frac{dN}{dt}=\lambda -\mu N-\left( {\delta }_{1}{I}_{m}+{\delta }_{2}{I}_{p} +{\delta }_{3}{I}_{mp}\right)$$

Since all the parameters are nonnegative, $\frac{dN}{dt}\le \lambda -\mu N$

Then, multiplying both sides using an integrating factor of $IF={e}^{\int \mu dt}={e}^{\mu t}$

$${e}^{\mu t}\left[\frac{dN}{dt}+\mu N\right]\le {e}^{\mu t}\lambda$$ $$\Rightarrow N\left(t\right)\le {{N}_{0}e}^{-\mu t}+\frac{\lambda }{\mu }$$ $$\Rightarrow$$ $$\underset{t\to \infty }{\mathrm{lim}}N\left(t\right)\le \frac{\lambda }{\mu }$$ Thus, $$0\le N\left(t\right)\le \frac{\lambda }{\mu }$$

There for the dynamical system is bounded.

#### Existence of the disease-free equilibrium point of the meningitis and pneumonia coinfections model

The disease-free equilibrium point of the system is determined by making all the equations in system (1) equal to zero, providing that $\{{I}_{p}={I}_{m}={I}_{mp}=0\}$ and become

$${{E}^{O}}_{mp}=\left({S}^{O},{{V}_{mp, }}^{O} {{I}_{p}}^{O},{{I}_{m, }}^{O} {{I}_{mp}}^{O}, {{T}_{mp}}^{O}{,R}^{O} \right)=\left(\frac{\lambda }{\mu }\left[\frac{\left(1-\pi \right)\left(\mu +\phi \right)+\uppi \phi }{\left(\upmu +\phi \right)}\right], \frac{\mathrm{\pi \lambda }}{\left(\upmu +\phi \right)},\mathrm{0,0},\mathrm{0,0},0\right)$$

#### Reproduction number

To compute the reproduction number, it is important to distinguish new infectious from all other changes in the host population. Let ${\mathcal{F}}_{i}\left(x\right):$ be the rate of appearance of new infectious in the compartment $i$ and ${{\mathcal{V}}^{+}}_{i}\left(x\right):$ be the rate of transfer of individuals in to the compartment $i,$ ${{\mathcal{V}}^{-}}_{i}\left(x\right): be$ the rate of transfer of individuals out of compartment $i$.

Then ${\mathcal{V}}_{i}(x)={ {\mathcal{V}}^{-}}_{i}\left(x\right)-{ {\mathcal{V}}^{+}}_{i}$ but $F=\left[\frac{\partial {\mathcal{F}}_{i}}{\partial {X}_{j}}({X}_{o})\right]$ and $V=\left[\frac{\partial {\mathcal{V}}_{i}}{\partial {X}_{j}}({X}_{o})\right]$, where $F$ and $V$ are $mxm$ matrices with $m$ being the number of infected compartment. $F{v}^{-1}$ is the next generation matrix, and the spectral radius of the next generation matrix is the needed reproduction number.

Thus, from our system we do have the following

$${\mathcal{F}}_{i}\left(x\right)=\left[\begin{array}{c}0\\ 0\\ {f}_{2}S\\ {f}_{1}S\\ 0\\ 0\\ 0\end{array}\right] \; and \; { \mathcal{V}}_{i}\left(x\right)=\left[\begin{array}{l} \left({f}_{1}+{f}_{2} +\mu \right)S-\left(1-\pi \right)\lambda -\rho R-\phi {V}_{mp} \\ \left(\upmu +\phi \right){V}_{mp}-\mathrm{\pi \lambda }\\ \left(\upomega{f}_{1} +{\tau }_{3} +{\delta }_{2} +\mu \right){I}_{p}\\ \left({\Theta f}_{2}+ {\tau }_{1}+{\delta }_{1}+\mu \right){I}_{m}\\ {-\upomega f}_{1}{I}_{p}-{\Theta f}_{2}{I}_{m}-\left({\tau }_{2}+\mu +{\delta }_{3}\right){I}_{mp}\\ \left(\beta +\mu \right){\mathrm{T}}_{mp}-{\tau }_{2}{I}_{mp}\\ \left(\rho +\mu \right)R-\beta {T}_{mp}-{\tau }_{1}{I}_{m}-{\tau }_{3}{I}_{P}\end{array}\right]$$

At disease free $N=S+{V}_{mp}$ and the infected compartment are $\left({I}_{p}, {I}_{m}, {I}_{mp}\right)$

Thus, $$N=S+{V}_{mp}=\frac{\lambda }{\mu }\left[\frac{\left(1-\pi \right)\left(\mu +\phi \right)+\uppi \phi }{\left(\upmu +\phi \right)}\right]+ \frac{\mathrm{\pi \lambda }}{\left(\upmu +\phi \right)}=\frac{\lambda \left(\left(1-\pi \right)\left(\mu +\phi \right)+\uppi \phi \right)+\mu \mathrm{\pi \lambda }}{\mu \left(\upmu +\phi \right)}=\frac{\uplambda }{\mu }$$

$$N=\frac{\uplambda }{\mu } and \frac{S}{N}=\frac{\left(1-\pi \right)\left(\mu +\phi \right)+\uppi \phi }{\left(\upmu +\phi \right)}$$

Thus $$F=\left[\begin{array}{ccc}{ \alpha }_{1}\frac{\lambda }{\mu }\left(\frac{\left(1-\pi \right)\left(\mu +\phi \right)+\uppi \phi }{\left(\upmu +\phi \right)}\right)& 0& { \alpha }_{1}\frac{\lambda }{\mu }\left(\frac{\left(1-\pi \right)\left(\mu +\phi \right)+\uppi \phi }{\left(\upmu +\phi \right)}\right)\\ 0& { \alpha }_{2}\frac{\lambda }{\mu }\left(\frac{\left(1-\pi \right)\left(\mu +\phi \right)+\uppi \phi }{\left(\upmu +\phi \right)}\right)& { \alpha }_{2}\frac{\lambda }{\mu }\left(\frac{\left(1-\pi \right)\left(\mu +\phi \right)+\uppi \phi }{\left(\upmu +\phi \right)}\right)\\ 0& 0& 0\end{array}\right]$$

$$V=\left[\begin{array}{ccc}{\tau }_{3} +{\delta }_{2} +\mu & 0& 0\\ 0& {\tau }_{1}+{\delta }_{1}+\mu & 0\\ 0& 0& {\tau }_{2}+\mu +{\delta }_{3}\end{array}\right]$$

Then $${FV}^{-1}=\left[\begin{array}{ccc}{ \alpha }_{1}\frac{\lambda }{\mu }\left(\frac{\left(1-\pi \right)\left(\mu +\phi \right)+\uppi \phi }{\left(\upmu +\phi \right)}\right)& 0& { \alpha }_{1}\frac{\lambda }{\mu }\left(\frac{\left(1-\pi \right)\left(\mu +\phi \right)+\uppi \phi }{\left(\upmu +\phi \right)}\right)\\ 0& { \alpha }_{2}\frac{\lambda }{\mu }\left(\frac{\left(1-\pi \right)\left(\mu +\phi \right)+\uppi \phi }{\left(\upmu +\phi \right)}\right)& { \alpha }_{2}\frac{\lambda }{\mu }\left(\frac{\left(1-\pi \right)\left(\mu +\phi \right)+\uppi \phi }{\left(\upmu +\phi \right)}\right)\\ 0& 0& 0\end{array}\right]\left[\begin{array}{ccc}\frac{1}{{\tau }_{3} +{\delta }_{2} +\mu }& 0& 0\\ 0& \frac{1}{{\tau }_{1}+{\delta }_{1}+\mu }& 0\\ 0& 0& \frac{1}{{\tau }_{2}+\mu +{\delta }_{3}}\end{array}\right]$$

$$G=\left[\begin{array}{ccc}{ \alpha }_{1}\frac{\lambda }{\mu }\left(\frac{\left(1-\pi \right)\left(\mu +\phi \right)+\uppi \phi }{({\tau }_{3} +{\delta }_{2} +\mu )\left(\upmu +\phi \right)}\right)& 0& { \alpha }_{1}\frac{\lambda }{\mu }\left(\frac{\left(1-\pi \right)\left(\mu +\phi \right)+\uppi \phi }{\left(\upmu +\phi \right)({\tau }_{2}+\mu +{\delta }_{3})}\right)\\ 0& { \alpha }_{2}\frac{\lambda }{\mu }\left(\frac{\left(1-\pi \right)\left(\mu +\phi \right)+\uppi \phi }{\left(\upmu +\phi \right)({\tau }_{1}+{\delta }_{1}+\mu )}\right)& { \alpha }_{2}\frac{\lambda }{\mu }\left(\frac{\left(1-\pi \right)\left(\mu +\phi \right)+\uppi \phi }{\left(\upmu +\phi \right)({\tau }_{2}+\mu +{\delta }_{3})}\right)\\ 0& 0& 0\end{array}\right]$$

Then, the corresponding eigenvalues of the next generation matrix G are

$${\lambda }_{1}={ \alpha }_{1}\frac{\lambda }{\mu }\left(\frac{\left(1-\pi \right)\left(\mu +\phi \right)+\uppi \phi }{({\tau }_{3} +{\delta }_{2} +\mu )\left(\upmu +\phi \right)}\right), {\lambda }_{2}=\left({ \alpha }_{2}\frac{\lambda }{\mu }\left(\frac{\left(1-\pi \right)\left(\mu +\phi \right)+\uppi \phi }{\left(\upmu +\phi \right)\left({\tau }_{1}+{\delta }_{1}+\mu \right)}\right)\right), {\lambda }_{3}=0$$

Therefore, the effective reproduction number is

$${\mathcal{R}}_{eff}=\mathrm{max}\left\{{ \alpha }_{1}\frac{\lambda }{\mu }\left(\frac{\left(1-\pi \right)\left(\mu +\phi \right)+\uppi \phi }{\left({\tau }_{3} +{\delta }_{2} +\mu \right)\left(\upmu +\phi \right)}\right), \left({ \alpha }_{2}\frac{\lambda }{\mu }\left(\frac{\left(1-\pi \right)\left(\mu +\phi \right)+\uppi \phi }{\left(\upmu +\phi \right)\left({\tau }_{1}+{\delta }_{1}+\mu \right)}\right)\right)\right\}.$$

The basic reproduction number that manipulated in the absence of an intervention is given by ${R}_{mp}=\mathrm{max}\left\{\frac{{ \lambda \alpha }_{1}}{\mu ({\tau }_{3} +{\delta }_{2} +\mu )} , \frac{{ \lambda \alpha }_{2}}{\mu \left({\tau }_{1}+{\delta }_{1}+\mu \right)}\right\}$

#### Local stability of the disease- free equilibrium point of the meningitis and pneumonia coinfection model

##### **Theorem 15**

*The disease-free equilibrium point* ${{E}^{O}}_{mp}=\left(\frac{\lambda }{\mu }\left[\frac{\left(1-\pi \right)\left(\mu +\phi \right)+\uppi \phi }{\left(\upmu +\phi \right)}\right], \frac{\mathrm{\pi \lambda }}{\left(\upmu +\phi \right)},\mathrm{0,0},\mathrm{0,0},0\right)$ *of the model in system* (1) *is locally asymptotically stable if the effective reproduction number* ${R}_{eff}<1$ *and unstable if* ${R}_{eff}>1$.

##### *Proof*

The Jacobean matrix $J\left({{E}^{O}}_{mp}\right)$ of the model (1) at the disease-free equilibrium point is

$$J\left({{E}^{O}}_{mp}\right)= \left[\begin{array}{ccccccc}-\mu & \phi & -{ \alpha }_{1}{n}_{1}& -{ \alpha }_{2}{n}_{1}& -({ \alpha }_{1}+{ \alpha }_{2}){n}_{1}& 0& \rho \\ 0& -\left(\upmu +\phi \right)& 0& 0& 0& 0& 0\\ 0& 0& { \alpha }_{1}{n}_{1}-{n}_{2}& 0& { \alpha }_{1}{n}_{1}& 0& 0\\ 0& 0& 0& { \alpha }_{1}{n}_{1}-{ n}_{3}& { \alpha }_{2}{n}_{1}& 0& 0\\ 0& 0& 0 & 0& {-n}_{4}& 0& 0\\ 0& 0& 0& 0& {\tau }_{2}& -\left(\beta +\mu \right)& 0\\ 0& 0& {\tau }_{3}& {\tau }_{1}& 0& \beta & -\left(\rho +\mu \right)\end{array}\right]$$

Then, the corresponding characteristic equation is given by

$$\left(-{\bigwedge }_{6}-(\beta +\mu )\right)\left(-{\bigwedge }_{2}-(\mu +\phi )\right)\left(-{\bigwedge }_{3}+({ \alpha }_{1}{n}_{1}-{n}_{2})\right)\left(-{\bigwedge }_{4}+({ \alpha }_{2}{n}_{1}-{n}_{3})\right)\left(-{\bigwedge }_{5}-{n}_{4}\right)(-\mu -{\bigwedge }_{1})(-\left(\rho +\mu \right)-{\bigwedge }_{7})=0$$

Thus, ${\bigwedge }_{1}=-\mu \; or \; {\bigwedge }_{2}=-\left(\mu +\phi \right) \; or \; {\bigwedge }_{3}=-\left({ \alpha }_{1}{n}_{1}-{n}_{2}\right) \; or \; {\bigwedge }_{4}=-({ \alpha }_{2}{n}_{1}-{n}_{3}) \; or \; {\bigwedge }_{5}=-{n}_{4} \; or \; {\bigwedge }_{6}=-(\beta +\mu ) \;or \;{\bigwedge }_{7}=-\left(\rho +\mu \right)$ Where ${n}_{1}=\frac{\lambda }{\mu }\left[\frac{\left(1-\pi \right)\left(\mu +\phi \right)+\uppi \phi }{\left(\upmu +\phi \right)}\right], {n}_{2}= \left( {\tau }_{3} +{\delta }_{2} +\mu \right),{ n}_{3}=\left( {\tau }_{1}+{\delta }_{1}+\mu \right)$ and ${n}_{4}= \left({\tau }_{2}+\mu +{\delta }_{3}\right)$, which are positive terms, all parameters are nonnegative, and $\pi$ is the proportion. Assuming that ${R}_{1}={ \alpha }_{1}\frac{\lambda }{\mu }\left(\frac{\left(1-\pi \right)\left(\mu +\phi \right)+\uppi \phi }{({\tau }_{3} +{\delta }_{2} +\mu )\left(\upmu +\phi \right)}\right)$ and ${R}_{2}={\alpha }_{2}\frac{\lambda }{\mu }\left(\frac{\left(1-\pi \right)\left(\mu +\phi \right)+\uppi \phi }{\left(\upmu +\phi \right)\left({\tau }_{1}+{\delta }_{1}+\mu \right)}\right)$ and by rearranging the term using the above assumption, we do have the following ${\bigwedge }_{3}=\left({{\tau }_{3} +{\delta }_{2} +\mu )(R}_{1}-1\right) \; and \; {\bigwedge }_{4}= \left({\tau }_{1}+{\delta }_{1}+\mu \right)\left({R}_{2}-1\right)$.

The roots ${\bigwedge }_{1}, {\bigwedge }_{2}, {\bigwedge }_{5}, {\bigwedge }_{6 } \; and \; {\bigwedge }_{7}$ are all less than zero. However the roots ${\bigwedge }_{3}=\left({{\tau }_{3} +{\delta }_{2} +\mu )(R}_{1}-1\right)$ and ${\bigwedge }_{4}= \left({\tau }_{1}+{\delta }_{1}+\mu \right)\left({R}_{2}-1\right)$ are negative if and only if ${R}_{1}<1$ and ${R}_{2}<1.$ Therefore, disease-free equilibrium point that is locally asymptotically stable if and only if ${R}_{eff}=\mathrm{max}\left\{{R}_{1}{, R}_{2}\right\}<1$ and it is unstable if ${R}_{eff}>1$.

#### Global stability of the disease-free equilibrium point of the meningitis and pneumonia coinfection only model

##### **Theorem 16**

*The disease-free equilibrium is globally asymptotically stable if* ${R}_{eff}=\mathrm{max}\left\{{R}_{1}{, R}_{2}\right\}<1$.

##### *Proof*

To prove the global asymptotic stability (G A S) of the disease- free equilibrium point, we used the Lyapunov function method. We defined a Lyapunov function assuming the coefficient of co-infected compartment is equal to zero as $L=a{I}_{m }+b{I}_{m }$ where $a=\frac{1}{{\tau }_{1}+{\delta }_{1}+\mu },\mathrm{ b}=\frac{1}{{\tau }_{3} +{\delta }_{2} +\mu }$ and then differentiating $L$ with respect to $t$

⇒ $\frac{dL}{dt}=\frac{{f}_{1}S}{{\tau }_{1}+{\delta }_{1}+\mu }+\frac{{f}_{2}S}{{\tau }_{3} +{\delta }_{2} +\mu }-{I}_{p}-{I}_{m}$. Then, by substituting the value of ${f}_{1} ={ \alpha }_{2}{I}_{m} , {f}_{2} ={ \alpha }_{1}{I}_{p}$ and $N=S+{V}_{mp}=\frac{\lambda }{\mu }$

$$\frac{dL}{dt}=\left[\frac{{ (\left(1-\pi \right)\left(\mu +\phi \right)+\uppi \phi )\alpha }_{2}}{({\tau }_{1}+{\delta }_{1}+\mu )\left(\upmu +\phi \right)}-1\right]{I}_{m}+\left[\frac{{ \alpha }_{1}\left(\left(1-\pi \right)\left(\mu +\phi \right)+\uppi \phi \right)}{{(\tau }_{3} +{\delta }_{2} +\mu )\left(\upmu +\phi \right)}-1\right]{I}_{p}$$

$\frac{dL}{dt}=\left({R}_{2}-1\right){I}_{m}+\left({R}_{1}-1\right){I}_{p}$ So $\frac{dL}{dt} \le 0$ if ${R}_{eff} \le 1$ and furthermore, $\frac{dL}{dt}=0$ if ${I}_{m} = 0$ and ${I}_{p}=0 .$ Holding these, we can see that $\left(\frac{\lambda }{\mu }\left[\frac{\left(1-\pi \right)\left(\mu +\phi \right)+\uppi \phi }{\left(\upmu +\phi \right)}\right], \frac{\mathrm{\pi \lambda }}{\left(\upmu +\phi \right)},0, 0, 0, 0, 0\right)$ is the only singleton in $\{\left(S, { V}_{m}, {I}_{m}, {I}_{p, } {I}_{mp, } {T}_{mp, } R\right)\in \Omega :\frac{dL}{dt}=0\}$. Therefore, by the principle of (LaSalle, 1976), DFE is globally asymptotically stable if ${R}_{eff}< 1$.

#### Existence of the endemic equilibrium point of the meningitis and pneumonia coinfection model

The endemic equilibrium point ${E}_{mp}^{*}=\left({S}^{*}, {{I}_{p}}^{*},{{I}_{m, }}^{*} {{I}_{mp}}^{*}, {{T}_{mp}}^{*}, {{V}_{mp, }}^{*}{,R}^{*}\right)$ exists when the disease persists in the community. From the analysis of the sub-model of meningitis only sub-model and the pneumonia only sub-model, we have shown that there is no endemic equilibrium point if ${\mathcal{R}}_{eff}<1$.

To find conditions necessary for the existence of the endemic equilibrium for ${\mathcal{R}}_{eff}>1$, the system of equations is equated to zero and solved equilibrium points in terms of the force of infections. After some algebraic manipulation, we do have an endemic equilibrium point

$${E}_{pm}^{*}=\left({S}^{*}, {{I}_{p}}^{*},{{I}_{m, }}^{*} {{I}_{mp}}^{*}, {{T}_{mp}}^{*}, {{V}_{mp, }}^{*}{,R}^{*}\right)$$

$${S}^{*}=\frac{\left(\pi -1\right)\lambda \left(\mu +\phi \right)-\phi \Pi \lambda }{\left(\mu +\phi \right)}\left[\frac{a}{\rho \left(\omega {\beta f}_{1}{f}_{2}{\tau }_{2}c+\Theta {\beta f}_{1}{f}_{2}{\tau }_{2}b+\left(\beta +\mu \right)\left(\mu +{\delta }_{3}+{\tau }_{2}\right)({f}_{2}{\tau }_{3}c+{f}_{1}{\tau }_{2}b)\right)-a\left({f}_{1}+{f}_{2} +\mu \right)}\right]$$

$${{I}_{p}}^{*}=\left(\frac{{f}_{2}}{\left(\upomega{f}_{1} +{\tau }_{3} +{\delta }_{2} +\mu \right)}\right){S}^{*}$$

$${{I}_{m }}^{*}=\frac{{f}_{1}{S}^{*}}{\left({\Theta f}_{2}+ {\tau }_{1}+{\delta }_{1}+\mu \right)}$$

$${{I}_{mp}}^{*}=\left(\frac{\upomega}{\left(\upomega{f}_{1} +{\tau }_{3} +{\delta }_{2} +\mu \right)}+\frac{\Theta}{\left({\Theta f}_{2}+ {\tau }_{1}+{\delta }_{1}+\mu \right)}\right)\frac{{f}_{2}{f}_{1}{S}^{*}}{\left({\tau }_{2}+\mu +{\delta }_{3}\right)}$$

$${{T}_{mp}}^{*}=\frac{{\tau }_{2}}{\left(\beta +\mu \right)}\left(\frac{\upomega}{\left(\upomega{f}_{1} +{\tau }_{3} +{\delta }_{2} +\mu \right)}+\frac{\Theta}{\left({\Theta f}_{2}+ {\tau }_{1}+{\delta }_{1}+\mu \right)}\right)\frac{{f}_{2}{f}_{1}{S}^{*}}{\left({\tau }_{2}+\mu +{\delta }_{3}\right)}$$

$${{V}_{mp }}^{*}=\frac{\mathrm{\Pi \lambda }}{\left(\upmu +\phi \right)}$$

$$\begin{aligned}{R}^{*}&=\frac{\beta {\tau }_{2}{f}_{2}{f}_{1}{S}^{*}}{\left(\rho +\mu \right)\left(\beta +\mu \right)\left({\tau }_{2}+\mu +{\delta }_{3}\right)}\left(\frac{\upomega}{\left(\upomega{f}_{1} +{\tau }_{3} +{\delta }_{2} +\mu \right)}+\frac{\Theta}{\left({\Theta f}_{2}+ {\tau }_{1}+{\delta }_{1}+\mu \right)}\right)\\&\quad+\frac{{\tau }_{1}{f}_{1}{S}^{*}}{\left(\rho +\mu \right)\left({\Theta f}_{2}+ {\tau }_{1}+{\delta }_{1}+\mu \right)}+\frac{{\tau }_{3}{f}_{2}{S}^{*}}{\left(\rho +\mu \right)\left(\upomega{f}_{1} +{\tau }_{3} +{\delta }_{2} +\mu \right)}\end{aligned}$$

where; $$a=\left(\beta +\mu \right)\left(\mu +\rho \right)\left(\mu +{\delta }_{3}+{\tau }_{2}\right)\left(\mu +{\delta }_{1}+{\Theta f}_{2}+{\tau }_{1}\right)\left(\mu +{\delta }_{3}+{\tau }_{2}+{\omega f}_{1}\right)$$

$$b=\mu +{\delta }_{3}+{\tau }_{2}+{\omega f}_{1},c=\mu +{\delta }_{1}+{\Theta f}_{2}+{\tau }_{1}$$

#### Local stability of the endemic equilibrium point of the meningitis and pneumonia coinfection model

##### **Theorem 17**

*The endemic equilibrium point* ${E}_{pm}^{*}=\left({S}^{*}, {{I}_{p}}^{*},{{I}_{m, }}^{*} {{I}_{mp}}^{*}, {{T}_{mp}}^{*}, {{V}_{mp, }}^{*}{,R}^{*}\right)$ *is locally stable when the effective reproduction number* ${\mathcal{R}}_{eff}>1$.

##### *Proof*

To show the local stability of the endemic equilibrium point, we use the Jacobian matrix and Routh Hurwitz stability criteria. Then, the Jacobian matrix of the dynamical system (1) is

$$J\left(X \right)=\left[\begin{array}{ccccccc}{A}_{1}& {A}_{2}& {A}_{4}& {A}_{5}& 0& \phi & \rho \\ {f}_{2}& {A}_{3}& {A}_{6}& {A}_{7}& 0& 0& 0\\ {f}_{1}& {A}_{8} & {A}_{9}& {A}_{10}& 0& 0& 0\\ 0& 0& 0& {B}_{1}& 0& 0& 0\\ 0& \upomega{f}_{1} & {\Theta f}_{2}& {\tau }_{2}& {B}_{2}& 0& 0\\ 0& 0& 0& 0& 0& {B}_{3}& 0\\ 0& {\tau }_{3}& {\tau }_{1}& 0& \beta & 0& {B}_{4}\end{array}\right]$$

where $${A}_{1}=-\left({f}_{1}+{f}_{2} +\mu \right), {A}_{2}=-{ \alpha }_{2}{S}^{*}, { {A}_{3}= \alpha }_{1}{S}^{*}-\left(\upomega{f}_{1} +{\tau }_{3} +{\delta }_{2} +\mu \right), {A}_{4}=-{ \alpha }_{1}{S}^{*}, {A}_{5}=-\left({ \alpha }_{1}+{ \alpha }_{2}\right){S}^{*}, {A}_{6}=-\upomega{ \alpha }_{2}{{I}_{p}}^{*}, {A}_{7}={ \alpha }_{1}{S}^{*}-\upomega{\alpha }_{2}{{I}_{p}}^{*}, {A}_{8}=-{ \alpha }_{1}\Theta{{I}_{m}}^{*}, {A}_{9}={ \alpha }_{2}{S}^{*}-\left({\Theta f}_{2}+ {\tau }_{1}+{\delta }_{1}+\mu \right),{A}_{10}={ \alpha }_{2}{S}^{*}-{ \alpha }_{1}\Theta{{I}_{m}}^{*},{B}_{1}=\upomega{{ \alpha }_{2}I}_{p}+{{ \alpha }_{1}\Theta I}_{m}-\left({\tau }_{2}+\mu +{\delta }_{3}\right), {B}_{2}=-\left(\beta +\mu \right),{B}_{3}=-\left(\upmu +\phi \right), {B}_{4}=-\left(\rho +\mu \right)$$

Then, the corresponding characteristic equation is given by

$${a}_{8}{\lambda }^{8}+{a}_{7}{\lambda }^{7}+{a}_{6}{\lambda }^{6}+{a}_{5}{\lambda }^{5}+{a}_{4}{\lambda }^{4}+{a}_{3}{\lambda }^{3}+{a}_{2}{\lambda }^{2}+{a}_{1}\lambda +{a}_{0}=0$$

where

$${a}_{8}=1, {a}_{7}=\left(-{A}_{1}-{A}_{3}-2{A}_{9}-{B}_{1}-{B}_{2}-{B}_{3}-{B}_{4}\right)$$

$$\begin{aligned}{a}_{6}&=\left({A}_{1}{A}_{3}+2{A}_{1}{A}_{9}+2{A}_{3}{A}_{9}+{A}_{9}^{2}+{A}_{1}{B}_{1}+{A}_{3}{B}_{1}+2{A}_{9}{B}_{1}+{A}_{1}{B}_{2}+{A}_{3}{B}_{2}+2{A}_{9}{B}_{2} +{B}_{1}{B}_{2}+{A}_{1}{B}_{3}\right. \\&\quad \left.+{A}_{3}{B}_{3}+2{A}_{9}{B}_{3}-{B}_{1}{B}_{3}-{B}_{2}{B}_{3}+{A}_{1}{B}_{4}+{A}_{3}{B}_{4}+2{A}_{9}{B}_{4}+{B}_{1}{B}_{4}+{B}_{2}{B}_{4}+{B}_{3}{B}_{4}\vphantom{{A}_{9}^{2}}\right)\end{aligned}$$

$$\begin{aligned}{a}_{5}&=\left({A}_{2}{f}_{2}-2{A}_{1}{A}_{3}{A}_{9}-{A}_{1}{A}_{9}^{2}-{A}_{3}{A}_{9}^{2}-{A}_{1}{A}_{3}{B}_{1}-2{A}_{1}{A}_{9}{B}_{1}-2{A}_{3}{A}_{9}{B}_{1}-{A}_{9}^{2}{B}_{1}-{A}_{1}{A}_{3}{B}_{2}\right.\\&\quad \left.-2{A}_{1}{A}_{9}{B}_{2}-2{A}_{3}{A}_{9}{B}_{2}-{A}_{9}^{2}{B}_{2}-{A}_{1}{B}_{1}{B}_{2}-{A}_{3}{B}_{1}{B}_{2}-2{A}_{9}{B}_{1}{B}_{2}-{A}_{1}{A}_{3}{B}_{3}-2{A}_{1}{A}_{9}{B}_{3}+\right.\\&\quad \left.+2{A}_{3}{A}_{9}{B}_{3}-{A}_{9}^{2}{B}_{3}-{A}_{1}{B}_{1}{B}_{3}-{A}_{3}{B}_{1}{B}_{3}-2{A}_{9}{B}_{1}{B}_{3}-{A}_{1}{B}_{2}{B}_{3}-{A}_{3}{B}_{2}{B}_{3}-2{A}_{9}{B}_{2}{B}_{3}\right.\\&\quad \left.-{B}_{1}{B}_{2}{B}_{3}-{A}_{1}{A}_{3}{B}_{4}-2{A}_{1}{A}_{9}{B}_{4}-2{A}_{3}{A}_{9}{B}_{4}-{A}_{9}^{2}{B}_{4}-{A}_{1}{B}_{1}{B}_{4}-{A}_{3}{B}_{1}{B}_{4}-2{A}_{9}{B}_{1}{B}_{4}\right.\\&\quad \left.-{A}_{1}{B}_{2}{B}_{4}-{A}_{3}{B}_{2}{B}_{4}-2{A}_{9}{B}_{2}{B}_{4}-{B}_{1}{B}_{2}{B}_{4}{-{A}_{1}{B}_{3}{B}_{4}-{A}_{3}{B}_{3}{B}_{4}-2{A}_{9}{B}_{3}{B}_{4}-{B}_{1}{B}_{3}{B}_{4}\lambda }^{5}-{B}_{2}{B}_{3}{B}_{4}+1\right)\end{aligned}$$

$$\begin{aligned}{a}_{4}&=\left({A}_{1}{A}_{3}{A}_{9}^{2}+2{A}_{1}{A}_{3}{A}_{9}{B}_{1}+{A}_{1}{A}_{9}^{2}{B}_{1}+{A}_{3}{A}_{9}^{2}{B}_{1}+2{A}_{1}{A}_{3}{A}_{9}{B}_{2}+{A}_{1}{A}_{9}^{2}{B}_{2}\right.\\&\quad\left.+{A}_{3}{A}_{9}^{2}{B}_{2}+{A}_{1}{A}_{3}{B}_{1}{B}_{2}+2{A}_{1}{A}_{9}{B}_{1}{B}_{2}+2{A}_{3}{A}_{9}{B}_{1}{B}_{2}+{A}_{9}^{2}{B}_{1}{B}_{2}+2{A}_{1}{A}_{3}{A}_{9}{B}_{3}\right.\\&\quad\left.+{A}_{1}{A}_{9}^{2}{B}_{3}+{A}_{3}{A}_{9}^{2}{B}_{3}+{A}_{1}{A}_{3}{B}_{1}{B}_{3}+2{A}_{1}{A}_{9}{B}_{1}{B}_{3}+2{A}_{3}{A}_{9}{B}_{1}{B}_{3}+{A}_{9}^{2}{B}_{1}{B}_{3}\right.\\&\quad\left.+{A}_{1}{A}_{3}{B}_{2}{B}_{3}+2{A}_{1}{A}_{9}{B}_{2}{B}_{3}+2{A}_{3}{A}_{9}{B}_{2}{B}_{3}+{A}_{9}^{2}{B}_{2}{B}_{3}+{A}_{1}{B}_{1}{B}_{2}{B}_{3}+{A}_{3}{B}_{1}{B}_{2}{B}_{3}\right.\\&\quad\left.+2{A}_{9}{B}_{1}{B}_{2}{B}_{3}+2{A}_{1}{A}_{3}{A}_{9}{B}_{4}+{A}_{1}{A}_{9}^{2}{B}_{4}+{A}_{3}{A}_{9}^{2}{B}_{4}+{A}_{1}{A}_{3}{B}_{1}{B}_{4}+2{A}_{1}{A}_{9}{B}_{1}{B}_{4}\right.\\&\quad\left.+2{A}_{3}{A}_{9}{B}_{1}{B}_{4}+{A}_{9}^{2}{B}_{1}{B}_{4}+{A}_{1}{A}_{3}{B}_{2}{B}_{4}+2{A}_{1}{A}_{9}{B}_{2}{B}_{4}+2{A}_{3}{A}_{9}{B}_{2}{B}_{4}+{A}_{9}^{2}{B}_{2}{B}_{4}\right.\\&\quad\left.+{A}_{1}{B}_{1}{B}_{2}{B}_{4}+{A}_{3}{B}_{1}{B}_{2}{B}_{4}+2{A}_{9}{B}_{1}{B}_{2}{B}_{4}+{A}_{1}{A}_{3}{B}_{3}{B}_{4}+2{A}_{1}{A}_{9}{B}_{3}{B}_{4}+2{A}_{3}{A}_{9}{B}_{3}{B}_{4}\right.\\&\quad\left.+{A}_{9}^{2}{B}_{3}{B}_{4}+{A}_{1}{B}_{1}{B}_{3}{B}_{4}+{A}_{3}{B}_{1}{B}_{3}{B}_{4}+2{A}_{9}{B}_{1}{B}_{3}{B}_{4}+{A}_{1}{B}_{2}{B}_{3}{B}_{4}+{A}_{3}{B}_{2}{B}_{3}{B}_{4}\right.\\&\quad\left.+2{A}_{9}{B}_{2}{B}_{3}{B}_{4}+{B}_{1}{B}_{2}{B}_{3}{B}_{4}-{A}_{2}{A}_{9}{f}_{2}-{A}_{2}{B}_{1}{f}_{2}-{A}_{2}{B}_{2}{f}_{2}-{A}_{2}{B}_{3}{f}_{2}-{A}_{2}{B}_{4}{f}_{2}\right.\\&\quad\left.-{A}_{2}{A}_{6}{f}_{1}-{A}_{3}-{B}_{1}-{B}_{2}-{B}_{3}-{B}_{4}+{A}_{4}^{2}{A}_{8}{f}_{1}{f}_{2}\right)\end{aligned}$$

$$\begin{aligned}{a}_{3}&=[2{A}_{1}{A}_{3}{A}_{9}{B}_{1}{B}_{4}-{A}_{1}{A}_{3}{A}_{9}^{2}{B}_{2}-2{A}_{1}{A}_{3}{A}_{9}{B}_{1}{B}_{2}-{A}_{1}{A}_{9}^{2}{B}_{1}{B}_{2}-{A}_{3}{A}_{9}^{2}{B}_{1}{B}_{2}\\&\quad-{A}_{1}{A}_{3}{A}_{9}^{2}{B}_{1}-{A}_{1}{A}_{3}{A}_{9}^{2}{B}_{3}-2{A}_{1}{A}_{3}{A}_{9}{B}_{1}{B}_{3}-{A}_{1}{A}_{9}^{2}{B}_{1}{B}_{3}-{A}_{3}{A}_{9}^{2}{B}_{1}{B}_{3}\\&\quad-2{A}_{1}{A}_{3}{A}_{9}{B}_{2}{B}_{3}-{A}_{1}{A}_{9}^{2}{B}_{2}{B}_{3}-{A}_{3}{A}_{9}^{2}{B}_{2}{B}_{3}-{A}_{1}{A}_{3}{B}_{1}{B}_{2}{B}_{3}-2{A}_{1}{A}_{9}{B}_{1}{B}_{2}{B}_{3}\\&\quad-2{A}_{3}{A}_{9}{B}_{1}{B}_{2}{B}_{3}-{A}_{9}^{2}{B}_{1}{B}_{2}{B}_{3}-{A}_{1}{A}_{3}{A}_{9}^{2}{B}_{4}-{A}_{1}{A}_{9}^{2}{B}_{1}{B}_{4}-{A}_{3}{A}_{9}^{2}{B}_{1}{B}_{4}\\&\quad-2{A}_{1}{A}_{3}{A}_{9}{B}_{2}{B}_{4}-{A}_{1}{A}_{9}^{2}{B}_{2}{B}_{4}-{A}_{3}{A}_{9}^{2}{B}_{2}{B}_{4}-{A}_{1}{A}_{3}{B}_{1}{B}_{2}{B}_{4}-2{A}_{1}{A}_{9}{B}_{1}{B}_{2}{B}_{4}\\&\quad-2{A}_{3}{A}_{9}{B}_{1}{B}_{2}{B}_{4}-{A}_{9}^{2}{B}_{1}{B}_{2}{B}_{4}-2{A}_{1}{A}_{3}{A}_{9}{B}_{3}{B}_{4}-{A}_{1}{A}_{9}^{2}{B}_{3}{B}_{4}-{A}_{3}{A}_{9}^{2}{B}_{3}{B}_{4}\\&\quad-{A}_{1}{A}_{3}{B}_{1}{B}_{3}{B}_{4}-2{A}_{1}{A}_{9}{B}_{1}{B}_{3}{B}_{4}-2{A}_{3}{A}_{9}{B}_{1}{B}_{3}{B}_{4}-{A}_{9}^{2}{B}_{1}{B}_{3}{B}_{4}-{A}_{1}{A}_{3}{B}_{2}{B}_{3}{B}_{4}\\&\quad-2{A}_{1}{A}_{9}{B}_{2}{B}_{3}{B}_{4}-2{A}_{3}{A}_{9}{B}_{2}{B}_{3}{B}_{4}-{A}_{9}^{2}{B}_{2}{B}_{3}{B}_{4}-{A}_{1}{B}_{1}{B}_{2}{B}_{3}{B}_{4}-{A}_{3}{B}_{1}{B}_{2}{B}_{3}{B}_{4}\\&\quad-2{A}_{9}{B}_{1}{B}_{2}{B}_{3}{B}_{4}+{A}_{2}{A}_{9}{B}_{1}{f}_{2}+{A}_{2}{A}_{9}{B}_{2}{f}_{2}+{A}_{2}{B}_{1}{B}_{2}{f}_{2}+{A}_{2}{A}_{9}{B}_{3}{f}_{2}+{A}_{2}{B}_{1}{B}_{3}{f}_{2}\\&\quad+{A}_{2}{B}_{2}{B}_{3}{f}_{2}+{A}_{2}{A}_{9}{B}_{4}{f}_{2}+{A}_{2}{B}_{1}{B}_{4}{f}_{2}+{A}_{2}{B}_{2}{B}_{4}{f}_{2}+{A}_{2}{B}_{3}{B}_{4}{f}_{2}-{A}_{2}{A}_{6}{B}_{1}{f}_{1}\\&\quad-{A}_{2}{A}_{6}{B}_{2}{f}_{1}-{A}_{2}{A}_{6}{B}_{3}{f}_{1}-{A}_{2}{A}_{6}{B}_{4}{f}_{1}+{A}_{3}{B}_{1}+{A}_{3}{B}_{2}+{B}_{1}{B}_{2}+{A}_{3}{B}_{3}+{B}_{1}{B}_{3}\\&\quad+{B}_{2}{B}_{3}+{A}_{3}{B}_{4}+{B}_{1}{B}_{4}+{B}_{2}{B}_{4}+{B}_{3}{B}_{4}-{A}_{4}^{2}{A}_{8}{B}_{1}{f}_{1}{f}_{2}-{A}_{4}^{2}{A}_{8}{B}_{2}{f}_{1}{f}_{2}\\&\quad-{A}_{4}^{2}{A}_{8}{B}_{3}{f}_{1}{f}_{2}-{A}_{4}^{2}{A}_{8}{B}_{4}{f}_{1}{f}_{2}\end{aligned}$$

$$\begin{aligned}{a}_{2}&={[A}_{1}{A}_{3}{A}_{9}^{2}{B}_{1}{B}_{2}+{A}_{1}{A}_{3}{A}_{9}^{2}{B}_{1}{B}_{3}+{A}_{1}{A}_{3}{A}_{9}^{2}{B}_{2}{B}_{3} +2{A}_{1}{A}_{3}{A}_{9}{B}_{1}{B}_{2}{B}_{3}+{A}_{1}{A}_{9}^{2}{B}_{1}{B}_{2}{B}_{3}\\ &\quad+{A}_{3}{A}_{9}^{2}{B}_{1}{B}_{2}{B}_{3} +{A}_{1}{A}_{3}{A}_{9}^{2}{B}_{1}{B}_{4}+{A}_{1}{A}_{3}{A}_{9}^{2}{B}_{2}{B}_{4}+2{A}_{1}{A}_{3}{A}_{9}{B}_{1}{B}_{2}{B}_{4}+{A}_{1}{A}_{9}^{2}{B}_{1}{B}_{2}{B}_{4}\\ &\quad+{A}_{3}{A}_{9}^{2}{B}_{1}{B}_{2}{B}_{4}+{A}_{1}{A}_{3}{A}_{9}^{2}{B}_{3}{B}_{4}+2{A}_{1}{A}_{3}{A}_{9}{B}_{1}{B}_{3}{B}_{4}+{A}_{1}{A}_{9}^{2}{B}_{1}{B}_{3}{B}_{4}+{A}_{3}{A}_{9}^{2}{B}_{1}{B}_{3}{B}_{4}\\ &\quad+2{A}_{1}{A}_{3}{A}_{9}{B}_{2}{B}_{3}{B}_{4}+{A}_{1}{A}_{9}^{2}{B}_{2}{B}_{3}{B}_{4}+{A}_{3}{A}_{9}^{2}{B}_{2}{B}_{3}{B}_{4}+{A}_{1}{A}_{3}{B}_{1}{B}_{2}{B}_{3}{B}_{4}+2{A}_{1}{A}_{9}{B}_{1}{B}_{2}{B}_{3}{B}_{4}\\ &\quad+2{A}_{3}{A}_{9}{B}_{1}{B}_{2}{B}_{3}{B}_{4}+{A}_{9}^{2}{B}_{1}{B}_{2}{B}_{3}{B}_{4}+{A}_{2}{A}_{9}{B}_{1}{B}_{2}{f}_{2}+{A}_{2}{A}_{9}{B}_{1}{B}_{3}{f}_{2}+{A}_{2}{A}_{9}{B}_{2}{B}_{3}{f}_{2}\\ &\quad+{A}_{2}{B}_{1}{B}_{2}{B}_{3}{f}_{2}+{A}_{2}{A}_{9}{B}_{1}{B}_{4}{f}_{2}+{A}_{2}{A}_{9}{B}_{2}{B}_{4}{f}_{2}+{A}_{2}{B}_{1}{B}_{2}{B}_{4}{f}_{2}+{A}_{2}{A}_{9}{B}_{3}{B}_{4}{f}_{2}\\ &\quad+{A}_{2}{B}_{1}{B}_{3}{B}_{4}{f}_{2}+{A}_{2}{B}_{2}{B}_{3}{B}_{4}{f}_{2}-{A}_{2}{A}_{6}{B}_{1}{B}_{2}{f}_{1}-{A}_{2}{A}_{6}{B}_{1}{B}_{3}{f}_{1}-{A}_{2}{A}_{6}{B}_{2}{B}_{3}{f}_{1}-{A}_{2}{A}_{6}{B}_{1}{B}_{4}{f}_{1}\\ &\quad-{A}_{2}{A}_{6}{B}_{2}{B}_{4}{f}_{1}-{A}_{2}{A}_{6}{B}_{3}{B}_{4}{f}_{1}-{A}_{3}{B}_{1}{B}_{2}-{A}_{3}{B}_{1}{B}_{3}-{A}_{3}{B}_{2}{B}_{3}-{B}_{1}{B}_{2}{B}_{3}-{A}_{3}{B}_{1}{B}_{4}\\ &\quad-{A}_{3}{B}_{2}{B}_{4}-{B}_{1}{B}_{2}{B}_{4}-{A}_{3}{B}_{3}{B}_{4}-{B}_{1}{B}_{3}{B}_{4}-{B}_{2}{B}_{3}{B}_{4}+{A}_{4}^{2}{A}_{8}{B}_{1}{B}_{2}{f}_{1}{f}_{2}+{A}_{4}^{2}{A}_{8}{B}_{1}{B}_{3}{f}_{1}{f}_{2}\\ &\quad+{A}_{4}^{2}{A}_{8}{B}_{2}{B}_{3}{f}_{1}{f}_{2}+{A}_{4}^{2}{A}_{8}{B}_{1}{B}_{4}{f}_{1}{f}_{2}+{A}_{4}^{2}{A}_{8}{B}_{2}{B}_{4}{f}_{1}{f}_{2}+{A}_{4}^{2}{A}_{8}{B}_{3}{B}_{4}{f}_{1}{f}_{2}]\end{aligned}$$

$$\begin{aligned}{a}_{1}&=\left({A}_{1}{A}_{3}{A}_{9}^{2}{B}_{1}{B}_{2}{B}_{4}-{A}_{1}{A}_{3}{A}_{9}^{2}{B}_{1}{B}_{2}{B}_{3}+-{A}_{1}{A}_{3}{A}_{9}^{2}{B}_{1}{B}_{3}{B}_{4}-{A}_{1}{A}_{3}{A}_{9}^{2}{B}_{2}{B}_{3}{B}_{4}\right.\\&\quad\left.-2{A}_{1}{A}_{3}{A}_{9}{B}_{1}{B}_{2}{B}_{3}{B}_{4}-{A}_{1}{A}_{9}^{2}{B}_{1}{B}_{2}{B}_{3}{B}_{4}-{A}_{3}{A}_{9}^{2}{B}_{1}{B}_{2}{B}_{3}{B}_{4}-{A}_{2}{A}_{9}{B}_{1}{B}_{2}{B}_{3}{f}_{2}\right.\\&\quad\left.-{A}_{2}{A}_{9}{B}_{1}{B}_{2}{B}_{4}{f}_{2}-{A}_{2}{A}_{9}{B}_{1}{B}_{3}{B}_{4}{f}_{2}-{A}_{2}{A}_{9}{B}_{2}{B}_{3}{B}_{4}{f}_{2}-{A}_{2}{B}_{1}{B}_{2}{B}_{3}{B}_{4}{f}_{2} \right.\\&\quad\left. +{A}_{3}{B}_{1}{B}_{2}{B}_{4}+{A}_{3}{B}_{1}{B}_{3}{B}_{4}+{A}_{3}{B}_{2}{B}_{3}{B}_{4}+{B}_{1}{B}_{2}{B}_{3}{B}_{4}-{A}_{4}^{2}{A}_{8}{B}_{1}{B}_{2}{B}_{3}{f}_{1}{f}_{2}\right.\\&\quad\left.-{A}_{4}^{2}{A}_{8}{B}_{1}{B}_{2}{B}_{4}{f}_{1}{f}_{2}-{A}_{4}^{2}{A}_{8}{B}_{1}{B}_{3}{B}_{4}{f}_{1}{f}_{2}-{A}_{4}^{2}{A}_{8}{B}_{2}{B}_{3}{B}_{4}{f}_{1}{f}_{2}\right.\\&\quad\left.-{A}_{2}{A}_{6}{B}_{1}{B}_{2}{B}_{3}{f}_{1}-{A}_{2}{A}_{6}{B}_{1}{B}_{2}{B}_{4}{f}_{1}-{A}_{2}{A}_{6}{B}_{1}{B}_{3}{B}_{4}{f}_{1}-{A}_{2}{A}_{6}{B}_{2}{B}_{3}{B}_{4}{f}_{1}\vphantom{{A}_{9}^{2}}\right)\end{aligned}$$

$${a}_{0}={A}_{1}{A}_{3}{A}_{9}^{2}{B}_{1}{B}_{2}{B}_{3}{B}_{4}-{A}_{2}{A}_{6}{B}_{1}{B}_{2}{B}_{3}{B}_{4}{f}_{1}+{A}_{2}{A}_{9}{B}_{1}{B}_{2}{B}_{3}{B}_{4}{f}_{2}+\lambda {A}_{3}{B}_{1}{B}_{2}{B}_{3}-{A}_{3}{B}_{1}{B}_{2}{B}_{3}{B}_{4}+{A}_{4}^{2}{A}_{8}{B}_{1}{B}_{2}{B}_{3}{B}_{4}{f}_{1}{f}_{2}$$

Hence, all the coefficients of the characteristic’s polynomial are positives if ${\mathcal{R}}_{eff}>1$.

By using the Routh-Hurwitz method, we obtained a Routh-Hurwitz array that had no sign change.

Therefore, the endemic equilibrium point of the meningitis and pneumonia co-dynamical system (1) is locally asymptotically stable.

#### Global stability of the endemic equilibrium point of the meningitis and pneumonia coinfection model

##### **Theorem 18 **

*If* ${R}_{eff}>1$, *the endemic equilibrium of the model * (1) *is globally asymptotically stable*.

##### *Proof*

Systematically, we define an appropriate Lyapunov function $L{^{\prime}}$ such that;

$$\begin{aligned}L{^{\prime}}&=\left(S-{S}^{*}+{S}^{*}ln\frac{{S}^{*}}{S}\right)+\left({I}_{m}-{{I}_{m}}^{*}+{{I}_{m}}^{*}ln\frac{{{I}_{m}}^{*}}{{I}_{m}}\right)+\left({I}_{p}-{{I}_{p}}^{*}+{{I}_{p}}^{*}ln\frac{{{I}_{p}}^{*}}{{I}_{p}}\right)\\&\quad+\left({I}_{mp}-{{I}_{mp}}^{*}+{{I}_{mp}}^{*}ln\frac{{{I}_{mp}}^{*}}{{I}_{mp}}\right)+\left({T}_{mp}-{{T}_{mp}}^{*}+{{T}_{mp}}^{*}ln\frac{{{T}_{mp}}^{*}}{{T}_{mp}}\right)\\ &\quad+\left({V}_{mp}-{{V}_{mp}}^{*}+{{V}_{mp}}^{*}ln\frac{{{V}_{mp}}^{*}}{{V}_{mp}}\right)+\left(R-{R}^{*}+{R}^{*}ln\frac{{R}^{*}}{R}\right)\end{aligned}$$

Then, after differentiating $L{^{\prime}}$ with respect to time $t$ and simplification

$$\Rightarrow \frac{dL{^{\prime}}}{dt}={Z}_{1}-{Z}_{2}$$ Where $${Z}_{1}= \lambda +\left({\Theta f}_{2}+ {\tau }_{1}+{\delta }_{1}+\mu \right){{I}_{m}}^{*}+\left(\upomega{f}_{1} +{\tau }_{3} +{\delta }_{2} +\mu \right){{I}_{p}}^{*}+\left({\tau }_{2}+\mu +{\delta }_{3}\right){{I}_{mp}}^{*}+\left(\beta +\mu \right){{T}_{mp}}^{*}+\left(\upmu +\phi \right){{V}_{mp}}^{*}+\left(\rho +\mu \right){R}^{*}$$

${Z}_{2}=\left(S\mu +{I}_{mp}\mu +{\mathrm{T}}_{mp}\mu +{I}_{m}\mu +{I}_{p}\mu +{V}_{mp}\upmu +R\mu +{I}_{mp}{\delta }_{3}+{{I}_{m}\delta }_{1}+{I}_{p}{\delta }_{2}+\left({f}_{1}+{f}_{2} +\mu \right){S}^{*}+\frac{{\left[\left(1-\pi \right)\lambda +\rho R+\phi {V}_{mp} \right]S}^{*}}{S}+\frac{{f}_{1}S{{I}_{m}}^{*}}{{I}_{m}}+\frac{\left[{\upomega f}_{1}{I}_{p}+{\Theta f}_{2}{I}_{m}\right]{{I}_{mp}}^{*}}{{I}_{mp}}+\frac{{{{f}_{2}SI}_{p}}^{*}}{{I}_{p}}+\frac{\left[{\tau }_{2}{I}_{mp}\right]{{T}_{mp}}^{*}}{{T}_{mp}}+\frac{{{\left[\mathrm{\pi \lambda }\right]V}_{mp }}^{*}}{{V}_{mp}}+\frac{\left[\beta {T}_{mp}+{\tau }_{1}{I}_{m}+{\tau }_{3}{I}_{P}\right]{R}^{*}}{R}\right)$*.* Thus, if ${Z}_{1} <{Z}_{2},$ then $\frac{dL}{dt}\le 0$, and $\frac{dL}{dt}=0$ if and only if $S={S}^{*}, {I}_{m}={{I}_{m}}^{*}, {I}_{p}={{I}_{p}}^{*}, {I}_{mp}={{I}_{mp}}^{*}, {T}_{mp}={{T}_{mp}}^{*}, { V}_{m}={{V}_{m }}^{*} \; and \; R={R}^{*}$

From this, we see that ${{E}^{*}}_{mp}=\left({S}^{*}, {{I}_{m}}^{*}, {{I}_{p}}^{*}, {{I}_{mp}}^{*}, {{T}_{mp}}^{*},{{V}_{m }}^{*}{, R}^{*}\right)$ is the largest compact invariant singleton set in $\left\{\left({S}^{*}, {{I}_{m}}^{*}, {{I}_{p}}^{*}, {{I}_{mp}}^{*}, {{T}_{mp}}^{*},{{V}_{m }}^{*}{, R}^{*}\right)\epsilon \Omega : \frac{{dL}^{{\prime}}}{dt}=0\right\}$

Therefore, by the principle of (LaSalle, 1976), the endemic equilibrium $({{E}^{*}}_{mp})$ is globally asymptotically stable in the invariant region if ${Z}_{1} <{Z}_{2}$.

## Sensitivity analysis of the model’s parameters

We carried out a sensitivity analysis to determine the model robustness to corresponding parameters values. If a variable is a differentiable function of the parameters, the sensitivity index may be alternatively defined using partial derivatives. Based on the definition of sensitivity analysis, we derive an analytical expression for the sensitivity of $({R}_{eff})$ as $\bigwedge {p}^{{R}_{eff}}=\left(\frac{\partial {R}_{eff}}{\partial \mathrm{p}}\right)\left(\frac{\mathrm{p}}{{R}_{o}}\right)$ to each of the parameters involved in $({R}_{eff})$. The most sensitive parameter are those whose sensitivity index magnitude larger than that of all other parameters. The sensitivity indices in terms of ${R}_{eff(p)}={\alpha }_{1}\frac{\lambda }{\mu }\left(\frac{\left(1-\pi \right)\left(\mu +\phi \right)+\uppi \phi }{({\tau }_{3} +{\delta }_{2} +\mu )\left(\upmu +\phi \right)}\right)$ with ${R}_{op}=\frac{{ \lambda \alpha }_{1}}{({\tau }_{3} +{\delta }_{2} +\mu )\mu }$ are stated in the Table:3 below.

In this study, we used the parameter values in Table 2 above and obtained ${\mathcal{R}}_{eff(p)}=11$ at ${\alpha }_{1}=0.959878$. The recruitment rate and pneumonia contract rate parameters has the highest impact on the basic reproduction number of pneumonia with absolute (0.99613 and 0.99614) respectively. The rate at which pneumonia infected individuals recovered naturally and pneumonia only caused death rate parameters are the least sensitive parameters. Here, sensitivity analysis shows that the most sensitive parameters are the pneumonia effective contact rate (${\alpha }_{1}$), the recruitment rate ($\lambda$). Moreover, the portion of vaccinated newborns ($\pi$) is the most negative sensitive parameter.

**Table 2:** Parameter values (NB: WHO’s 2019 relevant demographic data about Ethiopia are used).

No	Parameter	Parameter’s description	Value	Unit	Source
1	${\tau }_{1}$	The rate at which meningitis infected individual are recovered naturally	0.02	Time^−1^	^23^
2	${\tau }_{3}$	The rate at which pneumonia infected individual are recovered naturally	0.0115	Time^−1^	^23^
3	${\tau }_{2}$	The rate at which meningitis and pneumonia co infected individual treated and inter to treated class	0.3102	Time^−1^	^24^
4	$\beta$	The rate at which meningitis and pneumonia co infected individual are recovered after treatment (temporarily immunity to pneumonia and meningitis after treatment)	0.1	Time^−1^	^25^
5	$\mu$	Natural death rate	0.01	Time^−1^	^26^
6	${\delta }_{1}$	Meningitis only caused death rate	0.002–0.2	Time^−1^	^15^
7	${\delta }_{2}$	Pneumonia only caused death rate	0.006–0.5	Time^−1^	^26,27^
8	${\delta }_{3}$	Meningitis and pneumonia coinfection caused death rate	0.008–0.7	Time^−1^	^26^
9	$\omega$	Modification parameter and $\upomega \ge 1$	1	Time^−1^	Assumed
10	$\Theta$	The modification parameter and $\Theta\ge 1$	1	Time^−1^	Assumed
11	$\rho$	Rate of loss of immunity	0.00735–0.363	Time^−1^	^15^
12	$\pi$	The portion of vaccinated new born	0.105		^24,28^
13	$\lambda$	Recruitment rate	0.0413* N0	Size * time^−1^	WHO 2019
14	${\alpha }_{2}$	Meningitis contact rate	0.9	Size^−1^ * time^−1^	^27^
15	${\alpha }_{1}$	Pneumonia contract rate	0.007–0.6	Size^−1^ *time^−1^	^29^
16	$\phi$	Vaccine wanes rate	0.263	Time^−1^	^28,30^

The parameter’s values from different articles and WHO.

Using the parameter values in the Table 2, the sensitivity indices are stated for ${\mathcal{R}}_{eff(m)}$ in the table below (Table 4).

The sensitivity analysis shown in Table 4 elucidate that the most sensitive positive parameters is the meningitis contact rate $\left({\alpha }_{2}\right)$ and that the most sensitive negative parameter is the natural death rate ($\mu$) and the meningitis disease induced death rate (${\delta }_{1}$). These parameters have inverse relationships with the reproduction number, which means that smaller increases in these parameters will lead to a greater reduction in the basic reproduction number. Moreover, reduction in a smaller number of these parameters will cause a significant increase in the basic reproduction number.

Generally, the sign of each of the sensitive index values in Tables 3 and 4 indicates that a slight change in the parameters leads to change in the value of ${\mathcal{R}}_{eff(mp)}$. The ${\mathcal{R}}_{eff(mp)}=max{\{\mathcal{R}}_{eff(p)}, {\mathcal{R}}_{eff(m)}\}$ increases when sensitivity indices with positive signs increase, while ${\mathcal{R}}_{eff(mp)}=max {\{\mathcal{R}}_{eff(p)}, {\mathcal{R}}_{eff(m)}\}$ decreases when sensitivity indices with negative signs increase and vice versa.

**Table 3:** Sensitivity indices of ${\mathcal{R}}_{eff(p)}$.

Sensitivity index	Value
${\varvec{S}}{\varvec{I}}\left(\phi \right)$	0.003705
${\varvec{S}}{\varvec{I}}\left({\alpha }_{1}\right)$	0.99614
${\varvec{S}}{\varvec{I}}\left(\lambda \right)$	0.99613
${\varvec{S}}{\varvec{I}}\left(\pi \right)$	− 0.105
${\varvec{S}}{\varvec{I}}\left(\mu \right)$	0.0077899
${\varvec{S}}{\varvec{I}}\left({\tau }_{3}\right)$	− 0.0010599
${\varvec{S}}{\varvec{I}}\left({\delta }_{2}\right)$	− 0.001063

**Table 4:** Sensitivity indices of ${\mathcal{R}}_{eff(m)}$.

Sensitivity index	Value
${\varvec{S}}{\varvec{I}}\left({\alpha }_{2}\right)$	0.9961538
${\varvec{S}}{\varvec{I}}\left(\mu \right)$	− 0.8104
${\varvec{S}}{\varvec{I}}\left({\tau }_{1}\right)$	0.00067738
${\varvec{S}}{\varvec{I}}\left({\delta }_{1}\right)$	− 0.00099615

Biologically, the most sensitive parameters to ${\mathcal{R}}_{eff(p)}$ and ${\mathcal{R}}_{eff(m)}$ are found to be ${\alpha }_{1}$ and ${\alpha }_{2}$ respectively. These sensitive parameters can be controlled by means of interventions and preventions. Most specifically, the sensitivity indices ${\varvec{S}}{\varvec{I}}\left({\alpha }_{1} \right)=0.99614$ and ${\varvec{S}}{\varvec{I}}\left({\alpha }_{1} \right)=0.9961538$ mean that ${\mathcal{R}}_{eff(p)}$ or ${\mathcal{R}}_{eff(m)}$ approximately decreases by $0.99614$% and $0.9961538$% when ${\alpha }_{1}$ and ${\alpha }_{2}$ are decreased. This result will tell us, decrease in ${\alpha }_{1}$ and ${\alpha }_{2}$ is a possible intervention strategy for the reduction of ${\mathcal{R}}_{eff(mp)}$. A detail discussion on the changes in parameters ${\alpha }_{1}$ and ${\alpha }_{2}$ with their effects on ${\mathcal{R}}_{eff(p)}$ and ${\mathcal{R}}_{eff(m)}$ are held in the “Numerical simulation” section.

## Numerical simulations

The motivation behand consideration of numerical simulations are to study the behavior and rationality of mathematical models, which are too complex to provide analytical solutions, as in most nonlinear systems. The numerical results are manipulated for the dynamical system of the full meningitis and pneumonia coinfection model using the MATLAB numerical solver (ODE45). Since the accuracy and the speed at which the result of numerical computation of complicated system returned is faster, we were chosen ODE45. These simulations are done by using a set of parameter values in Table 2 above.

### The simulations of threshold

Mathematical models are constructed using the parameters that have biological representation and real-life situation. Additionally, the reproduction numbers that determine the stability of an equilibrium point are functions of these parameters. As the values of these parameters changes, there are Nemours biological changes and implications. In this part of our study, we simulated the dynamical system of the full model for various values of the associated reproduction thresholds ${\mathcal{R}}_{eff(m)}$ and ${\mathcal{R}}_{eff(p)}.$

#### **Case 1**

For $max \left\{{\mathcal{R}}_{{\varvec{e}}{\varvec{f}}{\varvec{f}}\left({\varvec{m}}\right)}\boldsymbol{ },{\mathcal{R}}_{{\varvec{e}}{\varvec{f}}{\varvec{f}}\left({\varvec{p}}\right)}\right\}<1$, (that is ${\mathcal{R}}_{{\varvec{e}}{\varvec{f}}{\varvec{f}}}<1$), the solution curves of the full meningitis and pneumonia coinfection model converge to the disease-free equilibrium point (DFE). This implies that the disease-free equilibrium point is locally asymptotically stable whenever the ${max\{ \mathcal{R}}_{{\varvec{e}}{\varvec{f}}{\varvec{f}}({\varvec{m}})}$ ${, \mathcal{R}}_{{\varvec{e}}{\varvec{f}}{\varvec{f}}({\varvec{p}})}\}<1$.

**Figure 2:**
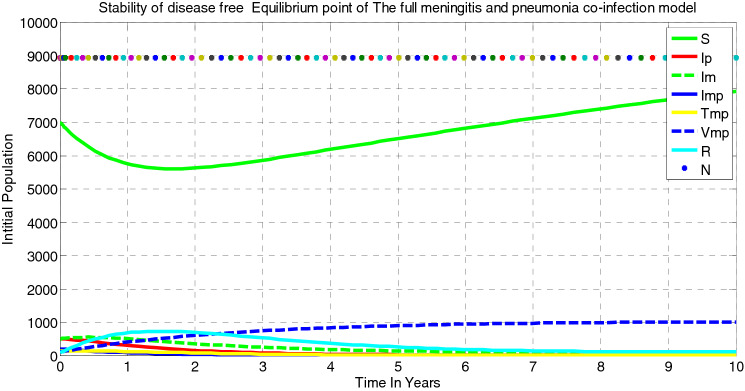
The stability of the disease-free equilibrium point (DFE).

Figure 2 above is plotted using the MATLAB ode45 program under consideration of reproduction numbers less than one. We can observe that all the solution curves of the system converge toward the disease-free equilibrium point. We obtained these results when the effective contact rate for pneumonia and meningitis are ${\alpha }_{1}=0.0001298$ and ${\alpha }_{2}=0.0001269$ which led to the effective reproduction number ${R}_{eff\left(p\right)}= 0.37 \; and \; {R}_{eff(m)}= 0.35$. At the disease-free equilibrium point, all the solution curves of the infection classes converge zero, while the susceptible and vaccinated classes increase exponentially for a long time, which indicates that the disease-free equilibrium point of the full pneumonia and meningitis coinfection model is globally asymptotically stable.

**Figure 3:**
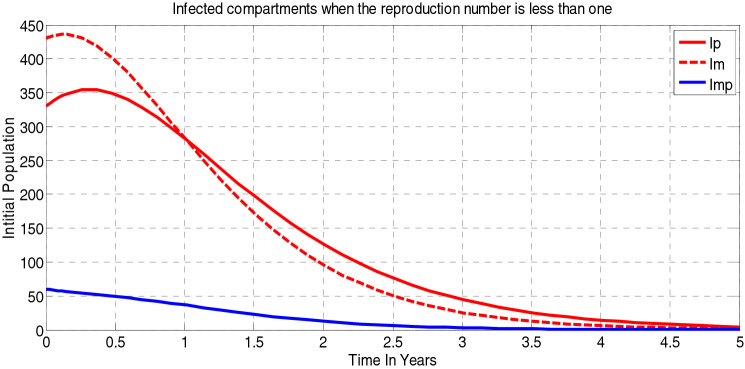
Infectious class when reproduction number is less than one.

From Fig. 3 shown above, we can see the behavior of the infectious classes for the full meningitis and pneumonia coinfection model when ${\mathcal{R}}_{eff(m)}<1$ and ${\mathcal{R}}_{eff(p)}<1$ that is all the infectious classes converge to the disease-free equilibrium point.

**Figure 4:**
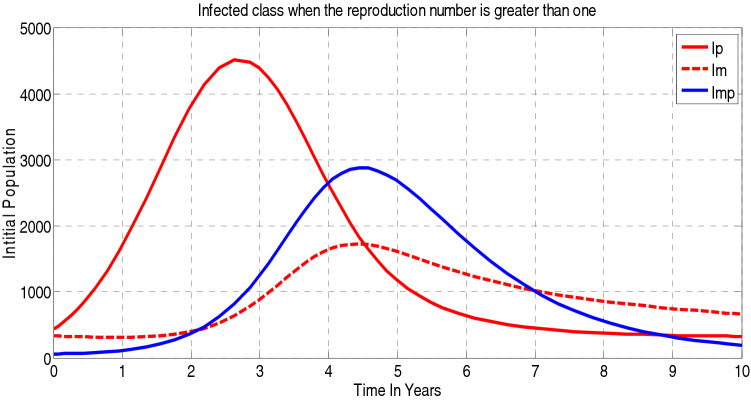
Infectious class when reproduction number is greater than one.

Figure 4 elucidate the physical properties of the solutions curves of the infectious classes of the full meningitis and pneumonia coinfection model for ${\mathcal{R}}_{eff(m)}>1$ and ${\mathcal{R}}_{eff(p)}>1$ which shows that all the solution curves are converge to the endemic equilibrium point.

#### **Case 2**

For $max \left\{{\mathcal{R}}_{{\varvec{e}}{\varvec{f}}{\varvec{f}}\left({\varvec{m}}\right)}\boldsymbol{ },\boldsymbol{ }\boldsymbol{ }{\mathcal{R}}_{{\varvec{e}}{\varvec{f}}{\varvec{f}}\left({\varvec{p}}\right)}\right\}>1$ (that is ${\mathcal{R}}_{{\varvec{e}}{\varvec{f}}{\varvec{f}}}>1$), the solution curves of the full meningitis and pneumonia coinfection model converge to the endemic equilibrium point (EE). This implies that the endemic equilibrium point (EE) is locally asymptotically stable whenever ${max\{ \mathcal{R}}_{{\varvec{e}}{\varvec{f}}{\varvec{f}}({\varvec{m}})}$ ${,\mathcal{R}}_{{\varvec{e}}{\varvec{f}}{\varvec{f}}({\varvec{p}})}\}>1$.

**Figure 5:**
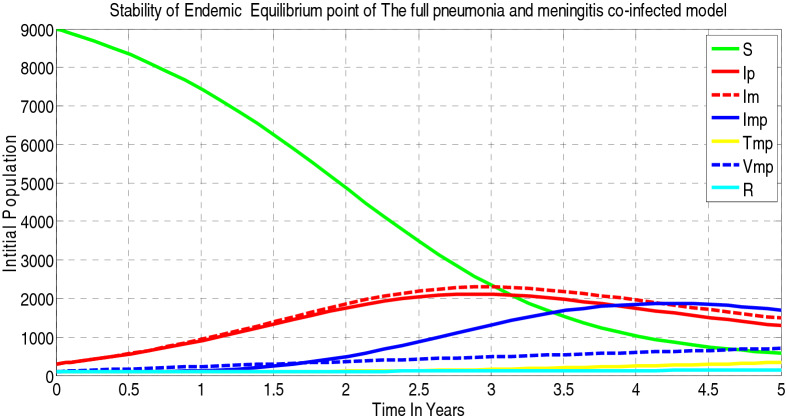
Stability of endemic equilibrium point of the meningitis and pneumonia confected model.

Figure 5 was plotted using the values of ${\mathcal{R}}_{eff(m)}=12.82$ with ${\alpha }_{1}=0.0001349$ and ${\mathcal{R}}_{eff(p)}=12.72$ with ${\alpha }_{2}=0.0001369$ which shows the stability of the endemic equilibrium point of the full meningitis and pneumonia coinfection model.

Additionally, we can observe that the effective reproduction number for pneumonia only infection is greater than the effective reproduction number of meningitis only infection. The plot shows that the endemic equilibrium point of the full meningitis and pneumonia coinfection model is globally asymptotically stable.

### Simulations of parameters with respect to the reproduction numbers

**Figure 6:**
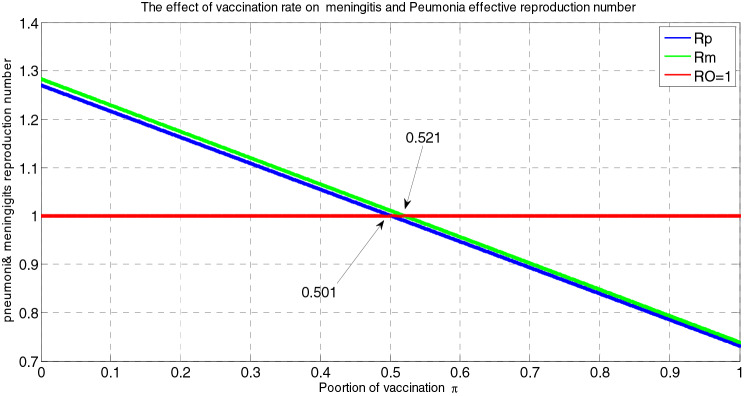
Effect of vaccination on meningitis and pneumonia effective reproduction number.

Figure 6 illustrated that pneumonia effective reproduction and meningitis effective reproduction number simulation at variable portion of vaccination and from the graph.

Biologically, we see that Pneumonia and meningitis infection dies out from the community when we apply the portion of vaccination with the rate $\pi >0.501$ and $\pi >0.521\mathrm{ f}$ or pneumonia and meningitis respectively.

**Figure 7:**
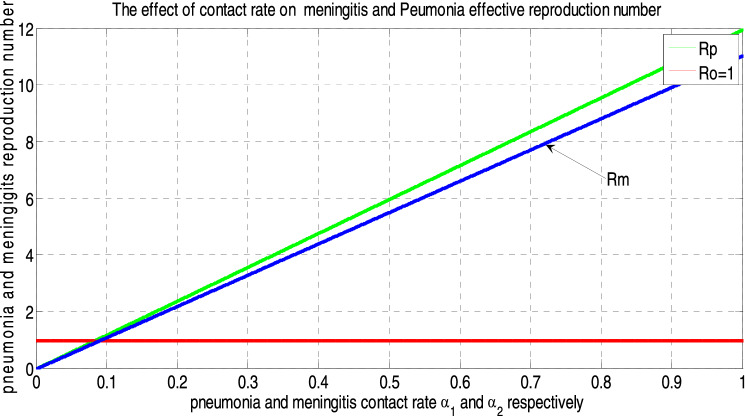
Effect of contact rate on meningitis and pneumonia effective reproduction number.

Figure 7 describe that pneumonia and meningitis infections die out from the community whenever the effective contact rate ${\alpha }_{1}<0.0074 \; and \; {\alpha }_{2}<0.0075$, respectively.

**Figure 8:**
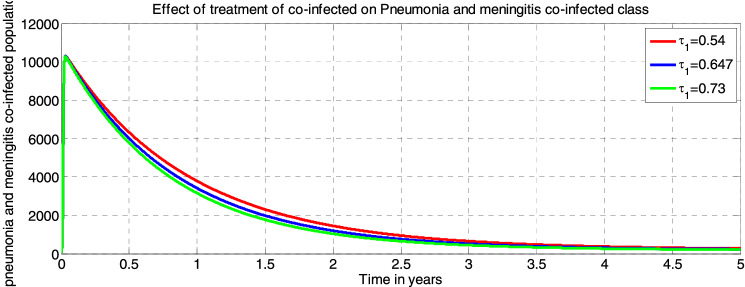
Effect of treatment of co-infected class on meningitis and pneumonia co-infected class.

**Figure 9:**
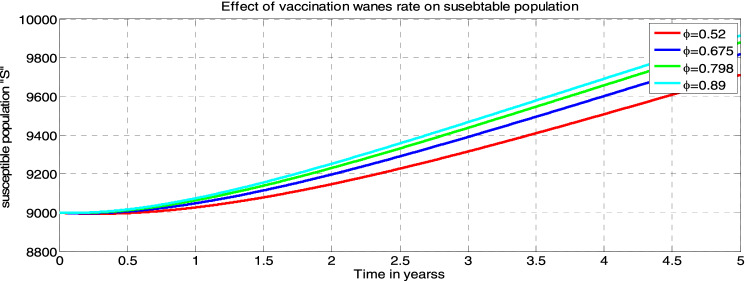
Effect of vaccination wanes on susceptible class.

Figure 8 shows the effective reproduction number of pneumonia and meningitis for different effective contact rates. Biologically, the plot describe how variations in treatment rates affect individuals who are co-infected with pneumonia and meningitis. The co-infected class decreased whenever the treatment rate increased, and the co-infected class increased as the treatment rate decreased. From Fig. 9 we can observe that as vaccine wanes increase, the susceptible populations also increase and vice versa.

**Figure 10:**
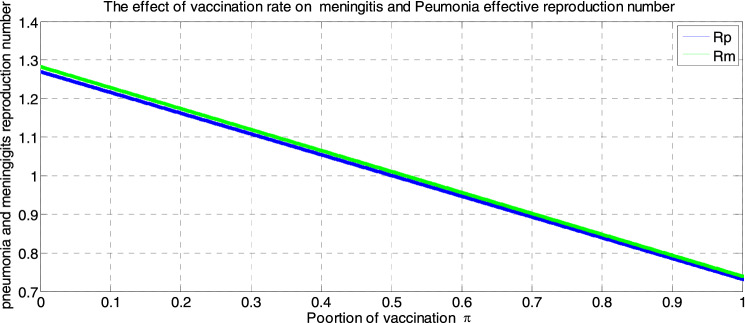
Effect of vaccination rate on meningitis and pneumonia reproduction number.

**Figure 11:**
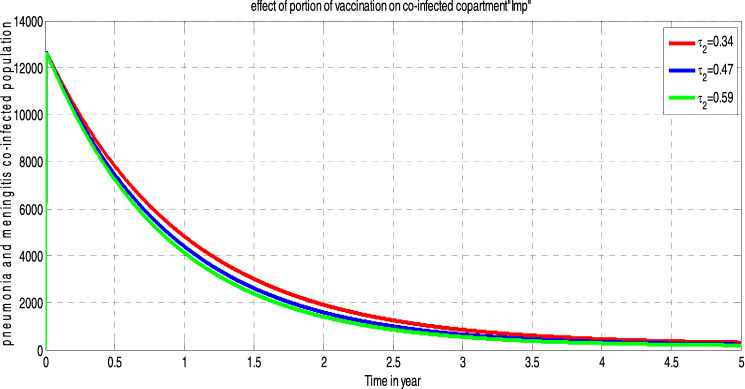
Effect of portion of vaccination on co-infected class.

Figure 10 shows that as the portion of vaccination of newly born individuals increases, the reproduction number of both diseases decreases. Figure 11 shows that as the portion of vaccination of newly born increases, the number of co-infected class decreases.

## Results and discussions

In this study, we formulated a mathematical model of seven nonlinear differential equations for the pneumonia and meningitis coinfection with PCV vaccination for a newly born population and treatment for co-infected class. We have considered the vaccination given for newborns called PCV13 (pneumococcal conjugate vaccine), which protects against numerous types of pneumococcal bacteria that can cause the most serious types of pneumococcal disease, including pneumonia and meningitis. We have also investigated the effect of treatment on the infection provided for the pneumonia and meningitis co-infected class. We have shown the positivity and boundedness of each sub model and fully pneumonia and meningitis coinfection model. The existence and uniqueness of disease free equilibrium point of each model, local stability and global stability of the disease free equilibrium points of the sub model and the full model are also investigated. We also analyzed the existence and uniqueness of the endemic equilibrium point of each model as well as the local stability and global stability of the endemic equilibrium points of each model. Our numerical simulation has shown that vaccination against those diseases, reducing contact with infectious persons and treatment have the great effect on reduction of these silent killer diseases from the communities.

Sub-Saharan Africa (i.e. which commonly known as Meningitis belt) including Ethiopia, are the region in trouble with meningitis disease. Most importantly meningitis, disease is a risk factor for pneumonia disease and vice versa. From these facts, we can say that the prevalence of pneumonia disease in this meningitis belt area is high.

## Conclusions and recommendations

Globally, all diseases, including meningitis and pneumonia require careful, continuous and constant nursing and medical attentions. One of the best and effective ways to control a pneumonia and meningitis diseases is to reduce contacts.

However, in our homeland Ethiopia with behavior and cultural value, a reduction in contact is not a successful prevention method. Vaccines and drugs are also common and recommendable ways that can potentially reduce the transmission of these diseases, which was the finding of our study. Additionally, hygiene has an important and mandatory role in the prevention of cerebrospinal meningitis (CSM) and pneumonia. Thus, persons should cover their noses and mouths while sneezing or coughing and discard used tissues promptly. Everyone should avoid smoking and exposure to secondhand smoke, which are risk factors for meningococcal disease. A person who closes contact with someone who has been diagnosed with these diseases may also need to take antibiotics, and symptoms relating to CSM and pneumonia should immediately be reported to the hospital for early treatment.

Our current results have some limitations, as they depend on the basic assumptions and there was a lack of literatures about meningitis and pneumonia co infection model. For further research, we recommended the development of a model that considers and holds additional protection and treatment that incorporates the awareness of our community.
